# C3AR1 may aggravate diabetic nephropathy by mediating oxidative stress via ITGB2 regulation in renal tubular epithelial cells

**DOI:** 10.1371/journal.pone.0331900

**Published:** 2025-09-12

**Authors:** Alex Abura, Shan Gao

**Affiliations:** Department of Endocrinology, Shaanxi Provincial People’s Hospital, Xi’an, China; Okayama University: Okayama Daigaku, JAPAN

## Abstract

Diabetes nephropathy (DN) is the most common chronic complication of diabetes and has become an important cause of end-stage renal failure. Oxidative stress and inflammatory response play important driving roles in the occurrence and development of diabetic nephropathy. As a key gene of DN, C3AR1 has been shown to mediate oxidative stress and inflammation. However, its potential mechanism in DN is still unclear. Here, we found that C3AR1 was upregulated in high glucose (HG)-treated human renal tubular epithelial cells (HK-2) and kidney tissues of DN rats. Interference with C3AR1 protected HK-2 cells from HG-mediated oxidative stress injury. Co-Immunoprecipitation (Co-IP) analysis showed that C3AR1 interacted with ITGB2 and promoted the expression of ITGB2. Overexpression of ITGB2 reversed the inhibition of C3AR1 interference on oxidative stress, apoptosis, and inflammatory response in HG-treated HK-2 cells. The rat DN model was established by unilateral nephrectomy and one-time intraperitoneal injection of 60 mg/kg streptozotocin (STZ), followed by the tail vein injection of the C3AR1 lentivirus interference plasmid. The results showed that interfering with C3AR1 reduced the level of inflammatory markers in the serum and weakened the oxidative stress and pathological damage of kidney tissues in DN rats. This study showed that C3AR1 may contribute to DN by upregulating ITGB2 protein levels to mediate oxidative stress.

## 1. Introduction

Diabetes mellitus is a disorder of carbohydrate, protein and fat metabolism caused by insufficient insulin secretion and/or insulin utilization disorder, with hyperglycemia as the main sign [1]. Diabetic nephropathy (DN) is one of the microvascular complications of diabetes. Renal disease caused by diabetes is the main cause of end-stage renal disease (ESRD) [2,3]. Its main features include changes in renal structure and function, such as glomerular hyperfiltration, microalbuminuria, glomerular basement membrane thickening, etc [4–6]. At present, DN is mainly treated by lifestyle changes, blood pressure control, blood glucose control and medication. However, lifestyle changes and blood pressure and blood glucose control can only delay the progression of DN disease to a certain extent and are ineffective in blocking the disease process, and the therapeutic efficacy of existing drugs is limited and has side effects [7–9]. Therefore, there is an urgent need to explore new molecular mechanisms of DN development and develop effective therapeutic strategies.

It is reported that DN develops into ESRD through a complex interaction of pathological processes such as chronic hyperglycemia, oxidative stress, and inflammation [10,11]. Among them, oxygen metabolism disorder is considered to be one of the mainly causes of DN kidney injury, which ultimately leads to harmful effects such as oxidative stress [12,13]. Oxidative stress leads to cell damage, elevated levels of transforming growth factor-β, microalbuminuria and glomerular cell apoptosis, and accelerates the progression of DN [10,11]. C3a anaphylatoxin chemotactic receptor (C3AR1) belongs to the G protein-coupled receptor 1 family, and its coding gene is located on chromosome 12 [14]. Reports have shown that C3AR1 is a key regulatory gene in metabolic diseases such as type 2 diabetes mellitus and fatty liver [15–17]. Chen et al. identified C3AR1 as a key regulator of IgA nephropathy by bioinformatics analysis [18]. Didier et al. reported that C3AR1 activation promotes renal fibrosis and progressive kidney disease [19]. Meanwhile, bioinformatics analysis by Hu et al. showed that C3AR1 was closely associated with diabetic nephropathy [20]. C3AR deficiency improves renal injury in diabetes by inhibiting inflammatory response [21]. C3AR antagonist ameliorates endothelial-myofibroblast transition and glomerular fibrosis in DN rats [22], and inhibits inflammation in type 2 diabetes nephropathy [23]. Therefore, we hypothesized that C3AR1 might be involved in the pathological process of DN. However, the molecular mechanism of C3AR1 in DN is unknown.

Integrins are a family of cell surface glycoproteins that sense the extracellular matrix and trigger a series of cellular responses to control cell adhesion, migration, proliferation, survival, and differentiation [24]. Recent evidence suggests that integrins also control cellular metabolism [25]. Integrin β2 (ITGB2) is a subunit of integrins, which is a heterodimeric surface receptor specifically expressed by leukocytes. It connects to the cytoskeleton and participates in intracellular signaling [24]. ITGB2 has been reported to be a potential diagnostic marker for DN [26,27]. In addition, Peng et al. reported that inhibition of ITGB2 attenuated epithelial mesenchymal transition and inflammatory responses in DN [28]. However, the molecular mechanisms by which ITGB2 is involved in DN disease progression is not fully understood.

In this study, we predicted and found that C3AR1 may interact with ITGB2 protein through the STRING database and verified it through Co-IP analysis. Next, human renal tubular epithelial cells (HK-2) treated with HG were used to construct an *in vitro* DN cell model, and gain-of-function and loss-of-function experiments for C3AR1 and ITGB2 were performed to explore their regulatory roles in DN. Finally, sh-sh-C3AR1 lentivirus was injected into DN rats induced by streptozotocin (STZ) via the tail vein *in vivo* to explore the effects of C3AR1 interference on pathological damage in DN rats. Our study aims to deepen the understanding of the pathogenesis of DN and provide new perspectives for the development of therapeutic strategies for DN.

## 2. Materials and methods

### 2.1. Cell culture

HK-2 cells (CL-0109) were purchased from Procell Life Science &Technology. HK-2 cells were cultured in DMEM/-12 (CAS: 11320033, Gibco, Grand Island, NY, USA) containing 10% FBS (CAS: A5670701, Thermo Fisher Scientific), 100 µg/mL streptomycin solution and penicillin (CAS: 15140122, Thermo Fisher Scientific) in a humidified atmosphere containing 5% CO_2_ at 37°C.

35 mM High glucose (HG) was used to treat HK-2 cells for 24 h to induce cell injury, and 5.5 mM glucose was used to treat HK-2 cells for 24 hours as control.

### 2.2. Cell transfection

40 and 80 nM C3AR1 siRNA was designed, synthesized, and validated by Thermo Fisher (USA). pcDNA-C3AR1, pcDNA-ITGB2 and pcDNA3.1 empty vector were purchased from Nanjing GenScript Biotech Corp. Subsequently, siRNAs (40 and 80 nM) and vectors (0.5 and 1.0 μg/mL) were transfected into the HK-2 cells for 48 and 72 h using Lipofectamine 3000 Reagent (Thermo Fisher, USA).

### 2.3. Quantitative real-time reverse transcription PCR (QPCR)

Total RNAs were extracted using the TRIzolTM reagent (15596018, Thermo Fisher) and reverse transcribed using the PrimeScript TM RT reagent Kit (TaKaRa, Dalian, China). Subsequently, TB Green ® Premix Ex Taq TM II (TaKaRa) was used for qPCR. The relative expression of genes was calculated using the 2^-ΔΔCt^ method, and *β-actin* was used as the control. The primer sequences are as follows: C3AR1, forward: 5’-CCC TAC GGC AGG TTC CTA TG-3’; reverse: 5’-GAC AGC GAT CCA GGC TAA TGG-3’; ITGB2, forward: 5’-TGC GTC CTC TCT CAG GAG TG-3’; reverse:5’- GGT CCA TGA TGT CGT CAG CC-3’; TNF-α, forward, 5’-TGA TCG GTC CCC AAA GGG ATG-3’, reverse: 5’-TTG GTG GTT T GC TAC GAC GTG G-3’; IL-6, forward: 5’-TGA TGC ACT TGC AGA AAA CAA TCT GA-3’, reverse: 5’-AGC TAT GGT ACT CCA GAA GAC CAG AGG-3’; IL-1β, forward, 5’-GCA ACT GTT CCT GAA CTC AAC T-3’, reverse: 5’-ATC TTT TGG GGT CCG TCA ACT-3’. *β-actin*, forward, 5’-TCG TGC GTG ACA TTA AGG AG-3’, reverse: 5’-GTC AGG CAG CTC GTA GCT CT-3’.

### 2.4. Western blot analysis

The cells were placed in cell lysis buffer and lysed on ice, and the total protein was extracted by centrifugation at 4°C. The concentration of the protein is calculated and quantified by the BCA Protein Assay Kit (Thermo Fisher). The protein was separated by 10% SDS-PAGE and transferred to a polyvinylidene fluoride membrane (Millipore, USA). The membrane was incubated with 5% skimmed milk powder for 2 hours. Subsequently, the membrane was incubated with anti-C3AR1 (1:500, ab126250, Abcam, Cambridge, UK), anti-ITGB2 (1:500, ab52920, Abcam) and anti-*β-actin* (1 µg/mL, ab213262, Abcam) antibodies overnight at 4°C. HRP-labeled secondary antibody IgG (1:100, ab6721, Abcam) was incubated with the membrane at room temperature for 2 h. Finally, the enhanced chemiluminescence kit (GE Healthcare, Chicago, IL, USA) was used to observe the immune response band, and the ImageJ software was used for optical density measurement and quantitative analysis.

### 2.5. MTT

Cell viability was detected by MTT kit (ml057897, Shanghai Enzyme Linked Biology). After a certain period of cell culture, 20 μL of MTT reagent was added. After incubation for 4h, 150 μL of DMSO reagent (Sigma Aldrich) was added. The absorbance was measured at 490 nm.

### 2.6. Reactive oxygen species (ROS) detection

The treated HK-2 cells were collected and the production of ROS was evaluated by ROS-sensitive fluorescent probe 5-(and-6)-chloromethyl-2’,7’-dichlorodihydrofluorescein, acetyl ester (CM-H2DCFDA) (Invitrogen, Life Technologies, Ltd.). Briefly, HK-2 cells (2 × 10^5^ cell/well) were incubated with 2’,7’-Dichlorodihydrofluorescein diacetate (DCFH-DA) fluorescent probe (10 μM/well) for 30 min at room temperature in the dark, and ROS production was detected by measuring the fluorescence intensity recorded at 495 nm excitation and 527 nm emission, using a microplate reader (Varian Cary Eclipse Fluorescence Spectrophotometer).

### 2.7. Determination of malondialdehyde (MDA) content

The content of MDA in HK-2 cells and rat renal tissues was determined by MDA content determination kit (ml077384, Shanghai Enzyme Linked Biology).

### 2.8. Nicotinamide Adenine Dinucleotide Phosphate Oxidase (NADPH) oxidase activity assay

NADPH activity in HK-2 cells was detected by colorimetry using NADPH detection kit (SNM006, Beijing Biolab).

### 2.9. Determination of Glutathione (GSH)

According to the manufacturer ‘s plan, GSH was quantified using a GSH assay kit (ml076450, Shanghai Enzyme linked Biological).

### 2.10. Superoxide Dismutase (SOD) activity detection

SOD activity was assessed using a SOD assay kit (ab65354, Abcam).

### 2.11. Cell apoptosis analysis

The apoptosis level of HK-2 was evaluated by using Annexin V/FITC and PI apoptosis detection kit (Becton Dickinson, USA). Briefly, HK-2 cells were collected, and were suspended in Annexin-binding buffer, followed by the staining with Annexin V- FITC/ PI for 15 min in darkness at room temperature. Subsequently, the stained cells were analyzed by using CYTOMICS FC 500 flow cytometer (Beckman Coulter, USA).

### 2.12. Construction of DN rat model

Eight-week-old SD rats (200-250g) were purchased from Xian Medical College. The rats were free to eat and drink, and raised at room temperature of 20–25°C with certain light. All rats were anesthetized by intraperitoneal injection of 1% (w/v) pentobarbital sodium (50 mg/kg, CAS: 9060-05-3, Chemical BooK, China), and a DN rat model was established by unilateral nephrectomy and one-time intraperitoneal injection of 60 mg/kg streptozotocin (STZ) as previously described [29]. Thirty-six rats were randomly divided into the control group, DN model group, and DN + sh-C3AR1 group, with 12 rats in each group. Rats in the sham controls received identical incision procedures without renal manipulation. One week post-surgery, when wounds had healed, DN model rats received intraperitoneal 60 mg/kg STZ injections, while rats in yhe sham controls were administered equivalent volumes of 0.1 M citrate buffer following overnight fasting. Rats in the DN + sh-C3AR1 group were injected with sh-C3AR1 lentivirus through the tail vein. After 72 h of STZ injection, the blood glucose of rats was measured by tail shear blood collection method. The blood glucose concentration higher than 16.8 mmol/mL was considered as successful modeling. Next, all rats in each group were euthanized with high-dose pentobarbital sodium (200 mg/kg, CAS: 9060-05-3, Chemical BooK, China), and kidney and serum samples were obtained. The experiment was conducted according to of the animal research laws of the National Institutes of Health Laboratory Animal Care, complied with the ARRIVE 2.0 committee guidelines, and was approved by the Ethics Committee of Shaanxi Provincial People’s Hospital (Approval number: SPPH-2024047).

### 2.13. H&E staining

After the rats were euthanized, the kidneys were taken out and fixed in a volume fraction of 10% formalin solution. After dehydration with gradient alcohol and xylene clarification, the kidneys were embedded in paraffin and cut into 5 μm thick tissue slices using a semi-automatic slicer. Subsequently, the tissue slices were subjected to conventional dewaxing, gradient ethanol hydration, hematoxylin staining for 10 min, and 1% eosin staining for 1 min. After washing with deionized water, they underwent gradient ethanol dehydration, xylene transparency, and neutral gum fixation. The tissue lesions were observed and photographed using an optical microscope.

### 2.14. Detection of blood urea nitrogen (BUN) and creatinine (Cr)

The supernatant was collected after 2 mL of rat blood was centrifuged. The BUN and Cr kits were used to detect the BUN content and Cr content of the rat serum.

### 2.15. Statistical analyses

SPSS 22.0 was used to analyze the data. Data distribution normality and homogeneity of variance were assessed using the Shapiro-Wilk test and Levene test, respectively. The data between two groups were analyzed using Student’s t test. One-way or two-way analysis of variance (ANOVA) was performed to assess statistical comparisons between multiple groups, followed by the least significant difference (LSD) test. The data are expressed as mean ± SD. *P* value < 0.05 was defined as statistically significant.

## 3. Results

### 3.1. C3AR1 is upregulated in HK-2 cells treated by HG

**Fig 1:**
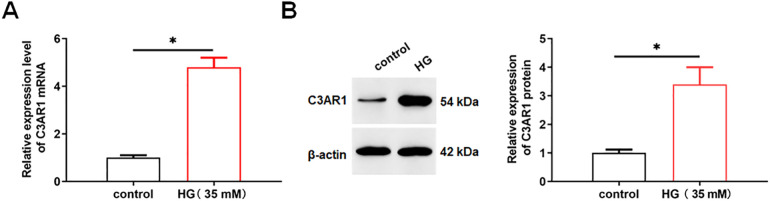
C3AR1 is upregulated in HG-treated HK-2 cells. HK-2 cells were treated with 5.5 mM glucose as control, and HK-2 cells were treated with 35 mM HG for 24 hours to establish a DN cell model. (A) QPCR was used to detect the expression of C3AR1 mRNA in HK-2 cells; (B) Western blotting was used to detect the expression level of C3AR1 protein in HK-2 cells. Data shown are the mean ± SD, N = 4. The statistical differences between two groups were analyzed using Student’s *t* test. Compared with the control group, *P < 0.05.

HK-2 cells were treated with 5.5 mM glucose or 35 mM High glucose (HG), and then the expression of C3AR1 was detected using qPCR and Western blotting. The results showed that C3AR1 were upregulated in HG treated HK-2 cells compared with normal glucose-treated cells (Fig 1A, 1B).

### 3.2. Interference with C3AR1 inhibits HG-mediated oxidative stress, apoptosis, and inflammatory response in HK-2 cells

**Fig 2:**
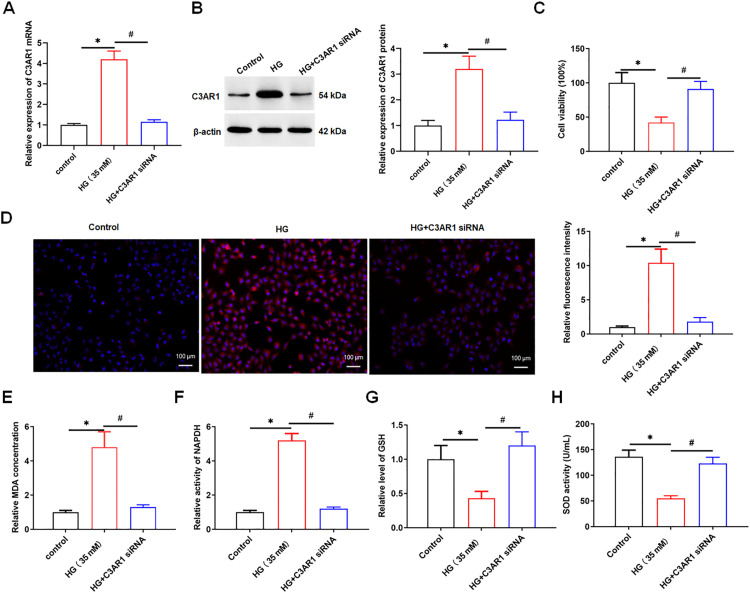
Interfering with C3AR1 inhibits HG-induced oxidative stress in HK-2 cells. HK-2 cells were treated with 35 mM HG for 24 hours to establish a DN cell model, and then 80 nM C3AR1 siRNA was transfected into HG-treated HK-2 cells for 48 hours. (A) QPCR was used to detect the expression of C3AR1 mRNA in HK-2 cells; (B) Western blotting was used to detect the expression level of C3AR1 protein in HK-2 cells; (C) HK-2 cell viability was detected by MTT assay; (D) DCFH-DA fluorescence probe method was used to detect lipid ROS level (original magnification, 100×), Scale bars = 1 cm; (E) MDA content detection kit to detect MDA content; (F) NADPH assay kit was used to analyze NADPH activity; (G) GSH detection kit to detect GSH concentration; (H) SOD detection kit to detect SOD activity. Data shown are the mean ± SD, N = 4. The statistical differences were evaluated by one-way ANOVA, and followed by LSD test. Compared with the control group, *P < 0.05; compared with the HG group, ^#^*P* < 0.05.

**Fig 3:**
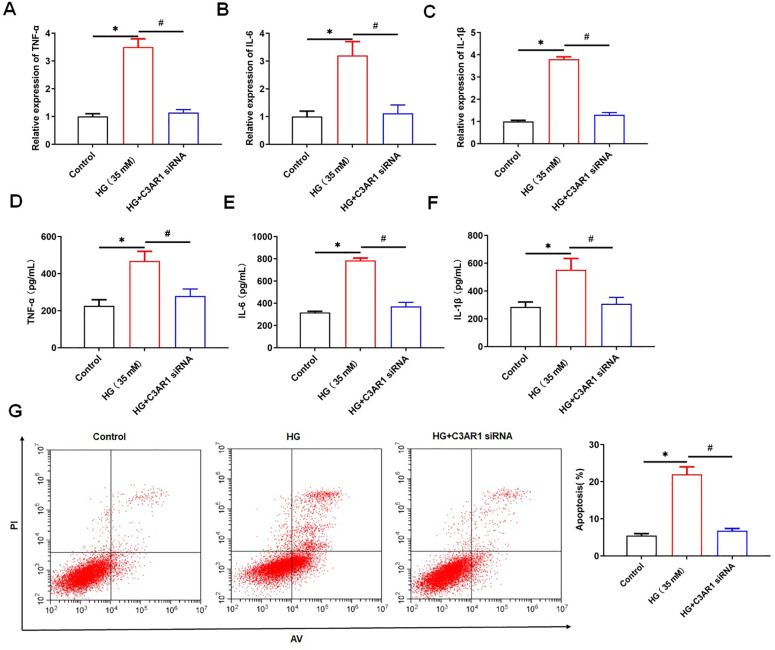
Interfering with C3AR1 inhibits HG-induced inflammation and apoptosis in HK-2 cells. HK-2 cells were treated with 35 mM HG for 24 hours to establish a DN cell model, and then were transfected with 80 nM C3AR1 siRNA for 48 hours. QPCR was used to detect the mRNA levels of inflammatory factor TNF-α (A), IL-6 (B), and IL-1β (C) in HK-2 cells; ELISA was used to detect the secretion level of inflammatory factor TNF-α (D), IL-6 (E), and IL-1β (F) in HK-2 cell culture supernatant; (G) Flow cytometry was used to detect the level of apoptosis. Data shown are the mean ± SD, N = 4. The statistical differences were evaluated by one-way ANOVA, and followed by LSD test. Compared with the control group, *P < 0.05, compared with the HG group, ^#^*P* < 0.05.

Next, we transfected C3AR1 siRNA into HG-treated HK-2 cells, the expression of C3AR1 was detected using qPCR and Western blotting. We found that C3AR1 was downregulated in HG-treated HK-2 cells after transfection of C3AR1 siRNA (Fig 2A, 2B). MTT results showed that HG treatment reduced the viability of HK-2 cells, while C3AR1 interference reversed this effect (Fig 2C). The ROS level, MDA content and NADPH activity in HK-2 cells were significantly increased after HG treatment, while GSH concentration and SOD activity were significantly decreased. Interfering with C3AR1 decreased the ROS level, MDA content and NADPH activity and increased GSH concentration and SOD activity in HG-treated HK-2 cells (Fig 2D-H). QPCR results showed that HG treatment promoted the mRNA expression of inflammatory factors TNF-α, IL-6 and IL-1β, while interference with C3AR1 decreased the mRNA levels of TNF-α, IL-6 and IL-1β in HG-treated HK-2 cells (Fig 3A-C). ELISA results showed that HG treatment increased the secretion levels of inflammatory factors TNF-α, IL-6 and IL-1β, while interference with C3AR1 reduced the secretion levels of TNF-α, IL-6 and IL-1β in HG-treated HK-2 cells (Fig 3D-F). The results of flow cytometry showed that HG treatment promoted the apoptosis of HK-2 cells, while interfering with C3AR1 reversed the promoting effect of HG on the apoptosis of HK-2 cells (Fig 3G). In summary, interference with C3AR1 inhibits HG-induced oxidative stress, apoptosis, and inflammatory response in HK-2 cells.

### 3.3. C3AR1 interacts with ITGB2

**Fig 4:**
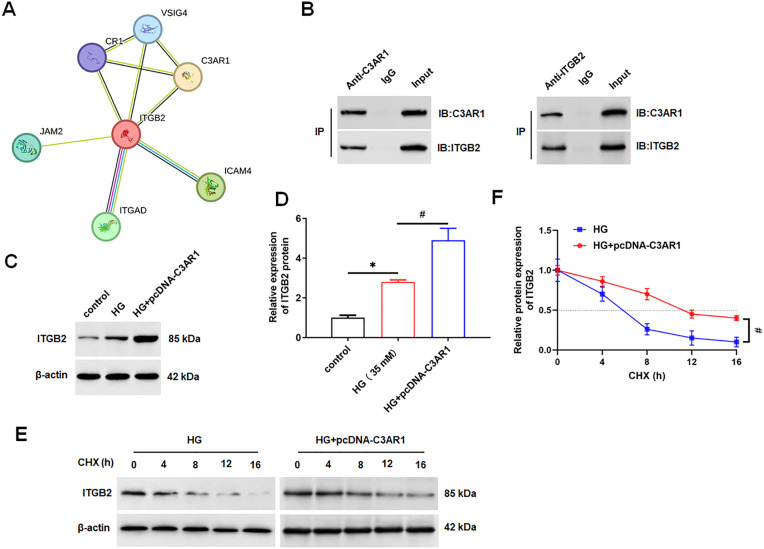
C3AR1 interacts with ITGB2 and promotes ITGB2 expression. (A) STRING was used to predict C3AR1 interacting proteins. (B) Co-IP detected the interaction between C3AR1 and ITGB2; (C) HK-2 cells were treated with 35 mM HG for 24 hours to establish a DN cell model, and then were transfected with 1.0 μg/mL pcDNA-C3AR1, and Western blotting was used to detect the expression level of ITGB2 protein in HK-2 cells; (D and E) After transfection of pcDNA-C3AR1 or empty vectors, HG-treated HK-2 cells were immediately treated with 5 μM cycloheximide (CHX) for 0, 4, 8, 12, or 16 hours, and Western blotting was used to detect the expression level of ITGB2 protein in HK-2 cells. Data shown are the mean ± SD, N = 4. The statistical differences were evaluated by one/two-way ANOVA, and followed by LSD test. Compared with the control group, *P < 0.05, compared with the HG group, ^#^*P* < 0.05.

In order to explore the molecular mechanism of C3AR1 regulating oxidative stress, the STRING tool was used to predict the interacting proteins of C3AR1, and ITGB2 was one of them (Fig 4A). The results of Co-IP experiments showed that C3AR1 and ITGB2 were co-precipitated (Fig 4B). ITGB2 was upregulated in HG-treated HK-2 cells, and overexpression of C3AR1 further upregulated the protein level of ITGB2 (Fig 4C and D). Therefore, we hypothesized that C3AR1 might affect the stability of ITGB2 protein. To verify our hypothesis, HK-2 cells transfected with pcDNA-C3AR1 or empty vectors were treated with cycloheximide (CHX), a protein synthesis inhibitor. The results showed that overexpression of C3AR1 significantly inhibited the degradation of ITGB2 protein (Fig 4E and F). The above results suggested that C3AR1 inhibits ITGB2 degradation by interacting with ITGB2, thereby upregulating ITGB2 protein levels.

### 3.4. C3AR1 promotes HG-induced oxidative stress and apoptosis in HK-2 cells by upregulating ITGB2 protein levels

**Fig 5:**
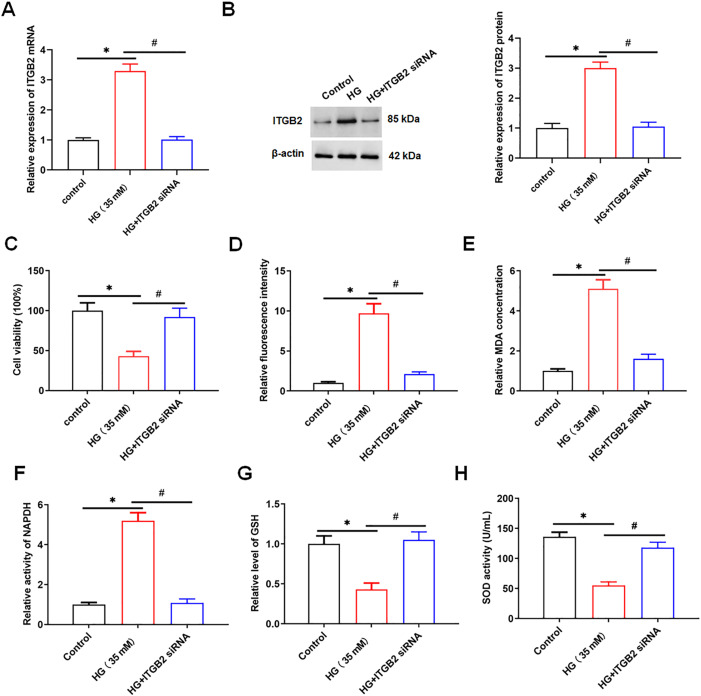
Interfering with ITGB2 inhibits HG-induced oxidative stress in HK-2 cells. HK-2 cells were treated with 35 mM HG for 24 hours to establish a DN cell model, and then 80 nM ITGB2 siRNA was transfected into HG-treated HK-2 cells for 48 hours. (A) QPCR was used to detect the expression of ITGB2 mRNA in HK-2 cells; (B) Western blotting was used to detect the expression level of ITGB2 protein in HK-2 cells; (C) HK-2 cell viability was detected by MTT assay; (D) DCFH-DA fluorescence probe method was used to detect lipid ROS level; (E) MDA content detection kit to detect MDA content; (F) NADPH assay kit was used to analyze NADPH activity; (G) GSH detection kit to detect GSH concentration; (H) SOD detection kit to detect SOD activity. Data shown are the mean ± SD, N = 4. The statistical differences were evaluated by one-way ANOVA, and followed by LSD test. Compared with the control group, *P < 0.05; compared with the HG group, ^#^*P* < 0.05.

**Fig 6:**
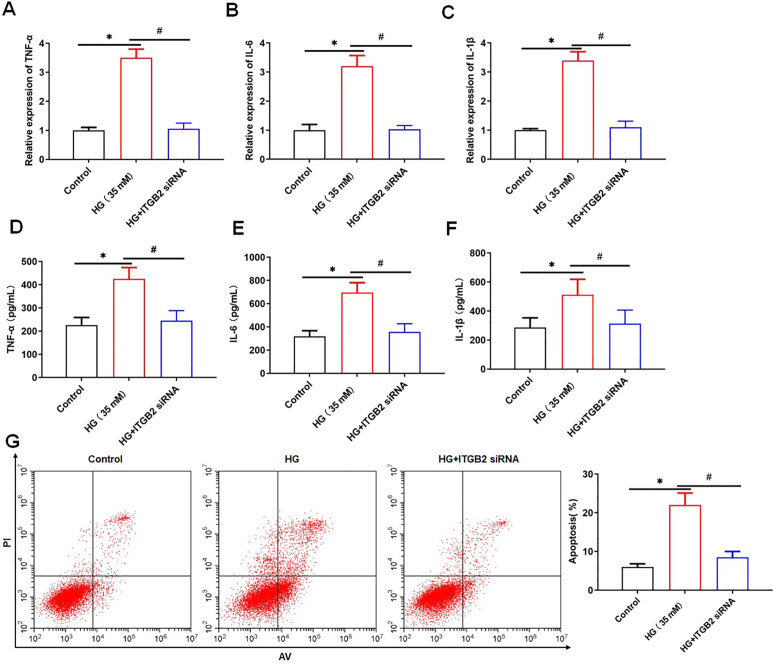
Interfering with ITGB2 inhibits HG-induced inflammation and apoptosis in HK-2 cells. HK-2 cells were treated with 35 mM HG for 24 hours to establish a DN cell model, and then were transfected with80 nM ITGB2 siRNA for 48 hours. QPCR was used to detect the mRNA levels of inflammatory factor TNF-α (A), IL-6 (B), and IL-1β (C) in HK-2 cells; ELISA was used to detect the secretion level of inflammatory factor TNF-α (D), IL-6 (E), and IL-1β (F) in HK-2 cell culture supernatant; (G) Flow cytometry was used to detect the level of apoptosis. Data shown are the mean ± SD, N = 4. The statistical differences were evaluated by one-way ANOVA, and followed by LSD test. Compared with the control group, *P < 0.05, compared with the HG group, ^#^*P* < 0.05.

To investigate the role of ITGB2 in DN, HG-treated HK-2 cells were transfected with ITGB2 siRNA and NC siRNA. The results showed that ITGB2 siRNA transfection significantly reduced ITGB2 mRNA and protein levels (Fig 5A and 5B). Moreover, ITGB2 interference increased cell viability, reduced ROS levels, MDA content, and NADPH activity, and increased GSH content and SOD activity in HG-treated HK-2 cells (Fig 5C-H). Meanwhile, interference with ITGB2 reduced the levels of inflammatory factors, such as TNF-α, IL-6, and IL-1β, and inhibited cell apoptosis in HG-treated HK-2 cells (Fig 6A-G). The above results indicated that interfering with ITGB2 inhibited oxidative stress, inflammatory response and cell apoptosis in HG-induced HK-2 cells.

**Fig 7:**
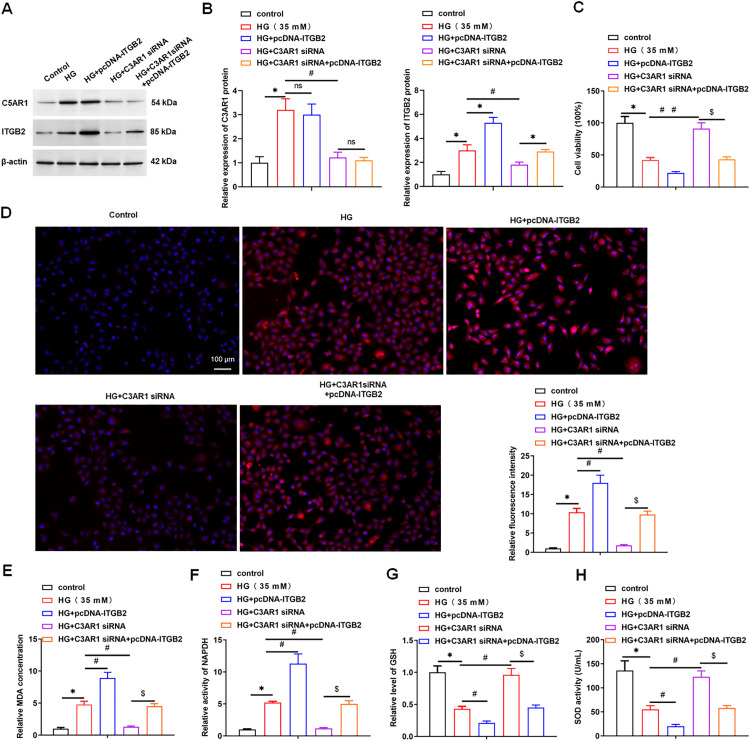
C3AR1 promotes HG-induced oxidative stress in HK-2 cells by upregulating ITGB2 protein level. HK-2 cells were treated with 35 mM HG for 24 hours to establish a DN cell model, and then were transfected with 80 nM C3AR1 siRNA or/and 1.0 μg/mL pcDNA-ITGB2 for 48 hours. (A and B) Western blotting was used to detect the expression level of C3AR1 protein in HK-2 cells; (C) HK-2 cell viability was detected by MTT assay; (D) DCFH-DA fluorescence probe method was used to detect lipid ROS level (original magnification, 100×), Scale bars = 1 cm; (E) MDA content detection kit to detect MDA content; (F) NADPH assay kit was used to analyze NADPH activity; (G) GSH detection kit to detect GSH concentration; (H) SOD detection kit to detect SOD activity. Data shown are the mean ± SD, N = 4. The statistical differences were evaluated by one-way ANOVA, and followed by LSD test. Compared with the control group, *P < 0.05, compared with the HG group, ^ns^*P* > 0.05, ^#^*P* < 0.05; compared with the HG + C3AR1 siRNA group, ^$^*P* < 0.05.

**Fig 8:**
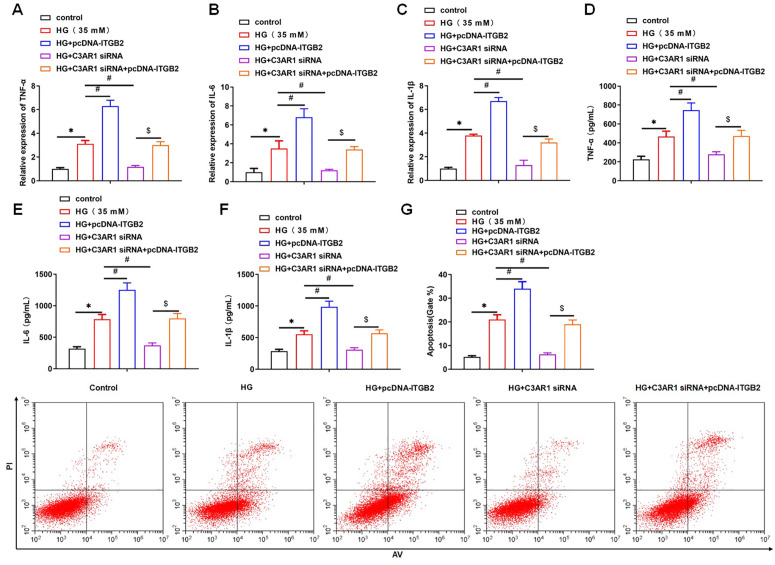
C3AR1 promotes HG-induced HK-2 cell inflammation and apoptosis by upregulating ITGB2 protein level. HK-2 cells were treated with 35 mM HG for 24 hours to establish a DN cell model, and then were transfected with 80 nM C3AR1 siRNA or/and 1.0 μg/mL pcDNA-ITGB2 for 48 hours. QPCR was used to detect the mRNA levels of inflammatory factor TNF-α (A), IL-6 (B), and IL-1β (C) in HK-2 cells; ELISA was used to detect the secretion level of inflammatory factor TNF-α (D), IL-6 (E), and IL-1β (F) in HK-2 cell culture supernatant; (G) Flow cytometry was used to detect the level of apoptosis. Data shown are the mean ± SD, N = 4. The statistical differences were evaluated by one-way ANOVA, and followed by LSD test. Compared with the control group, *P < 0.05, compared with the HG group, ^#^*P* < 0.05; compared with the HG + C3AR1 siRNA group, ^$^*P* < 0.05.

Then, we explored whether C3AR1 affected the oxidative stress of HK-2 cells by promoting the expression of ITGB2. The results showed that interference with C3AR1 inhibited C3AR1 and ITGB2 expression, while overexpression of ITGB2 increased ITGB2 protein levels, but had no significant effect on C3AR1 expression (Fig 7A, 7B). MTT results showed that interference with C3AR1 improved the reduction of cell viability by HG treatment, while overexpression of ITGB2 offset the promoting effect of C3AR1 interference on HG-treated cell viability (Fig 7C). Interfering with C3AR1 decreased ROS level, MDA content and NADPH activity in HG-treated HK-2 cells, and increased GSH concentration and SOD activity. Overexpression of ITGB2 reversed the effect of C3AR1 interference on oxidative stress in HG-treated HK-2 cells (Fig 7D-H). The results showed that interference with C3AR1 reduced the inflammatory response in HK-2 cells, which was manifested by the decrease of mRNA expression and secretion levels of inflammatory factors (TNF-α, IL-6 and IL-1β) in cells, while overexpression of ITGB2 reversed the inhibition of C3AR1 interference on the inflammatory response in HK-2 cells (Fig 8A-F). The results of flow cytometry showed that interference with C3AR1 inhibited the apoptosis of HK-2 cells treated with HG, while overexpression of ITGB2 reversed the inhibition of C3AR1 interference on the apoptosis of HK-2 cells (Fig 8G). Meanwhile, we found that interference with C3AR1 reversed the effects of ITGB2 overexpression on oxidative stress, inflammatory response and apoptosis in HG-induced HK-2 cells in a dose- and time-dependent manner (Supplementary Figure 1). In addition, we also explored the effects of C3AR1 and ITGB2 overexpression on non-diabetic kidney disease, and the results showed that overexpression of C3AR1 increased C3AR1 and ITGB2 expression, while overexpression of ITGB2 increased ITGB2 protein levels, but had no significant effect on C3AR1 expression (Supplementary Figure 2). Furthermore, overexpression of both C3AR1 and ITGB2 promoted oxidative stress and inflammation and induced apoptosis in HK-2 cells (Supplementary Figure 3). The above results suggested that C3AR1 might promote oxidative stress, inflammatory response and apoptosis in HK-2 cells by upregulating ITGB2 protein levels, especially under HG conditions, where its promoting effect is more pronounced.

### 3.5. Interference with C3AR1 potentially ameliorates DN in rats by inhibiting oxidative stress

**Fig 9:**
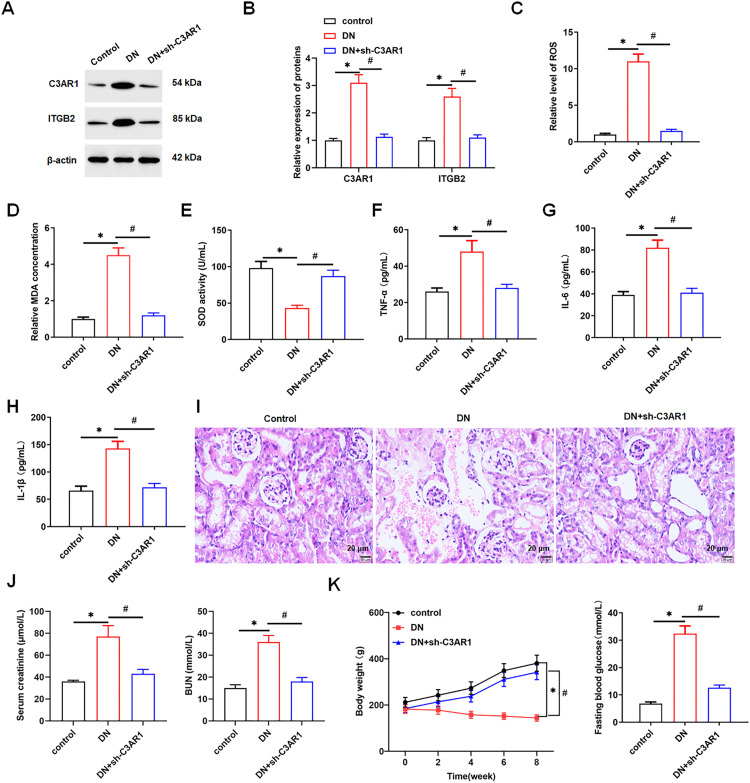
Interfering with C3AR1 potentially ameliorates diabetic nephropathy in rats by inhibiting oxidative stress. Thirty-six rats were randomly divided into the control group, DN model group, and DN + sh-C3AR1 group, with 12 rats in each group. The rat DN model was established by unilateral nephrectomy and one-time intraperitoneal injection of 60 mg/kg STZ. Rats in the DN + sh-C3AR1 group were administrated sh-C3AR1 lentivirus via the tail vein after the DN model was established. (A and B) Western blotting was used to detect the expression levels of C3AR1 and ITGB2 proteins in rat kidney tissues; (C) DCFH-DA fluorescent probe method was used to detect the level of lipid ROS in renal tissues; (D) MDA content detection kit to detect MDA content in renal tissues; (E) SOD detection kit was used to detect the activity of SOD in renal tissues; ELISA was used to detect the secretion level of inflammatory factor TNF-α (F), IL-6 (G), and IL-1β (H) in rat serum; (I) H&E staining was used to analyze renal tissue damage (original magnification, 200×), Scale bars = 0.4 cm; (J) Detection of serum creatinine and urea levels in rats; (K) body weight and fasting blood glucose of rats. Data shown are the mean ± SD, N = 4. The statistical differences were evaluated by one/two-way ANOVA, and followed by LSD test. Compared with the control group, *P < 0.05; compared with DN group, ^#^*P* < 0.05.

Finally, we verified the results of the *in vitro* cell experiments through *in vivo* rat experiments. The results showed that the proteins of C3AR1 and ITGB2 were highly expressed in kidney tissues of DN rats compared with the control group. Interference with C3AR1 decreased the protein levels of C3AR1 and ITGB2 in the kidney tissues of DN rats (Fig 9A and 9B). Moreover, the ROS level and MDA content were higher and the SOD activity was lower in the kidney tissue of DN rats compared to the control group, and interference with C3AR1 decreased the ROS level and MDA content and increased the SOD activity in the kidney tissue of DN rats (Fig 9C-E). The results of ELISA showed that the secretion levels of inflammatory factors (TNF-α, IL-6 and IL-1β) in the serum of DN rats were higher, while interference with C3AR1 reduced the secretion levels of TNF-α, IL-6 and IL-1β in the serum of DN rats (Fig 9F-H). The results of H&E staining showed that the kidney tissue of control rats were morphologically and structurally intact and clearly visible; whereas DN model rats had severe renal dysfunction, mainly manifested in glomerular and tubulointerstitial damage and inflammatory lymphocyte infiltration; the interference with C3AR1 reduced inflammatory lymphocyte infiltration and alleviated the pathological damage of the kidney tissues in DN rats (Fig 9I). Compared with the control group, the levels of Cr and BUN in the serum of DN model rats were increased, while interference with C3AR1 decreased the levels of Cr and BUN in the serum of DN model rats (Fig 9J). In addition, the body weight of DN rats was also significantly reduced and the blood glucose level was significantly increased compared with the control group. Interfering with C3AR1 elevated the body weight and decreased blood glucose levels in DN model rats (Fig 9K). The above results indicated that interfering with C3AR1 could potentially ameliorate pathological damage in DN rats by inhibiting oxidative stress.

## 4. Discussion

At present, the treatment of DN is mainly achieved by the combination of blood glucose control and blood pressure control, lifestyle changes and pharmacologic interventions. However, anti-hyperglycemic agents and anti-hypertensive therapies do not easily eliminate the inflammatory response, and their pathogenesis is complex and progressive renal damage is difficult to correct [4,8,30]. And therapeutic drugs have some degree of side effects [8,31]. Therefore, DN treatment is still challenging. In this study, we explored the potential molecular mechanism of C3AR1 affecting DN. Our results showed that C3AR1 may promote oxidative stress, apoptosis, and inflammatory response by upregulating ITGB2 protein levels, thereby aggravating the pathological features of DN.

Oxidative stress is considered to be the key pathway of DN under metabolic abnormalities [32]. In addition, inflammation and renal tubular cell apoptosis can also be used as the cause and typical sign of DN pathology [33,34]. Evidence has shown that HG-mediated oxidative stress and inflammatory response promote the development and progression of DN by promoting apoptosis and other pathological changes in cells [35]. Research has found that carotenoids improve DN in mice by reducing oxidative stress [36]. Telmisartan attenuates DN by reducing oxidative stress and inflammation in diabetic rats [37]. TXNIP aggravates DN by mediating oxidative stress and NLRP3 inflammasome activation [38]. Dioscin alleviates DN by inhibiting oxidative stress and apoptosis [39]. In addition, it has been reported that ChREBP deficiency prevents HG-induced HK-2 cell apoptosis by inhibiting oxidative stress, thereby improving renal function in DN mice [40]. Sabrina et al. reported that urinary biomarkers of renal tubular injury (u-NGAL, u-B2M, u-OPN, u-TFF3, and u-Cys) were increased in hyperbilirubinemic patients [41]. Consistent with these reports, our results showed that interference with C3AR1 inhibited oxidative stress, inflammation, and apoptosis in HK-2 cells and reduced the levels of urinary biomarkers of tubular injury in DN rats, thereby attenuating renal impairment in DN rats.

As a destructive neuroinflammatory factor, C3AR1 is a main regulator of microglial neuroinflammatory function, and its deletion reduces the risk of degeneration in mice with high intraocular pressure [42]. A study has found that C3AR1 is highly expressed in tissues around the hematoma and may affect the process of cerebral hemorrhage through neuroinflammation and apoptosis [43]. The expression of C3AR1 is positively correlated with inflammatory response and mediates oxidative stress [44]. C3AR1 depletion inhibits oxidative stress in microglia and mitigates HIF-1α-induced metabolic damage, thereby ameliorating cognitive dysfunction in Alzheimer’s disease [45]. In the present study we found that interference with C3AR1 alleviated renal functional injury in DN rats by inhibiting HG-induced oxidative stress, inflammation and apoptosis in HK-2 cells. Interestingly, other reports found that C3AR1 exerts anti-inflammatory protective effects in inflammation-related diseases. For example, Brennan et al. reported that during the acute inflammatory response triggered by traumatic spinal cord injury, C3AR1 binds to PTEN to block the PI3K/AKT pathway, thereby inhibiting CXCR2-driven bone marrow neutrophil mobilization/recruitment and reducing inflammatory pathological damage [46]. Tampe et al. reported that the expression level of C3AR1 in glomeruli was positively correlated with renal function recovery after treatment in patients with systemic lupus erythematosus-associated nephritis, particularly in stage III lupus nephritis [47]. Boos et al. reported that C3A exerts a protective anti-inflammatory effect via C3AR in LPS-induced sepsis mice [48]. The above results indicated that C3AR1 plays a dual role in inflammation-related diseases, which may be related to the stage of disease onset.

Further explore the molecular mechanism of C3AR1 in DN, we found that C3AR1 interacts with ITGB2. Integrin β2 (ITGB2), a subunit of β2 integrin, has been reported as a potential diagnostic marker for DN [26,27]. Therefore, we hypothesized that C3AR1 might be involved in DN progression by regulating ITGB2. In addition, ITGB2 has been found to be closely associated with immune cell infiltration in a variety of inflammation-related diseases. For example, Xu et al. reported that inhibiting ITGB2 alleviates inflammatory responses by blocking the recruitment of resting dendritic cells, thereby alleviating inflammatory bowel disease in mice [49]. Zhu et al. reported that ITGB2 participates in chronic sinusitis by regulating M2 macrophages [50]. Li et al. reported that interfering with ITGB2 inhibits macrophage adhesion to vascular endothelial cells by reducing the secretion of inflammatory factors and chemokines, thereby alleviating myocardial hypertrophy in mice [51]. Meanwhile, Peng et al. reported that inhibition of ITGB2 attenuated epithelial mesenchymal transition and inflammatory responses in HG-treated HK-2 cells [28]. In the present study we found a phenomenon similar to the above reports, overexpression of ITGB2 enhanced the inflammatory response and oxidative stress and promoted apoptosis in HG-induced HK-2 cells.

In this study, we revealed for the first time the role of the C3AR1/ITGB2 axis in DN progression. However, this study still has some limitations. First, the regulation of renal function is a complex process, which may involve the participation of multiple entity cells and immune cells, as well as the regulation of multiple molecular mechanisms, whether C3AR1 and ITGB2 are involved in DN progression by participating in immune regulation and other signaling pathways is not clear. We used lentiviral vectors of C3AR1 shRNA to knockdown C3AR1 in DN rats for *in vivo* validation experiments, and it remains to be demonstrated how the lentiviral vectors injected via the tail vein are targeted and whether there is an off-target effect. In addition, the role of ITGB2 in DN rats and whether the role of C3AR1 in DN rats is achieved through the regulation of ITGB2 also need to be further verified in subsequent studies.

In summary, we demonstrated that C3AR1 promoted renal injury and inflammatory factor secretion in DN rats by upregulating ITGB2 protein levels to promote oxidative stress, thereby aggravating the pathological features of DN. These findings provide a new perspective for the development of therapeutic strategies for DN.

## Supporting information

S1 Fig C3AR1 promotes HG-induced oxidative stress, inflammation and apoptosis in HK-2 cells through dose-dependent upregulation of ITGB2 protein levels. HK-2 cells were treated with 5.5 mM glucose as control, and HK-2 cells were treated with 35 mM HG for 24 hours to establish a DN cell model, and then transfected with C3AR1 siRNA (40 and 80 nM) or/and pcDNA-ITGB2 (0.5 and 1.0 μg/mL) for 48 and 72 hours. (A-C) Western blotting was used to detect the expression level of C3AR1 protein in HK-2 cells; (D) HK-2 cell viability was detected by MTT assay; (E) MDA content detection kit to detect MDA content; (F) SOD detection kit to detect SOD activity; (G) QPCR was used to detect the expression of inflammatory factor TNF-α mRNA in HK-2 cells; (H) QPCR was used to detect the expression of inflammatory factor IL-6 mRNA in HK-2 cells; (I) QPCR was used to detect the expression level of inflammatory factor IL-1β mRNA in HK-2 cells; (J) Flow cytometry was used to detect the level of apoptosis. Data shown are the mean ± SD, N = 4. The statistical differences were evaluated by one-way or two-way ANOVA, and followed by LSD test. Compared with the control group, the HG group, the HG + pcDNA-ITGB2 (1.0 μg/mL) group, ns *P* > 0.05, *P < 0.05, **P < 0.01.
(TIF)

S2 Fig Overexpression efficiency of C3AR1 and ITGB2 in HK-2 cells. HK-2 cells were transfected with pcDNA-C3AR1 (1.0 μg/mL) or pcDNA-ITGB2 (1.0 μg/mL) for 48 hours. (A) Western blotting was used to detect the protein levels of C3AR1 (B) and ITGB2 (C). Data shown are the mean ± SD, N = 4. The statistical differences were evaluated by one-way ANOVA, and followed by LSD test. Compared with the control group, the pcDNA-vector group, ns *P* > 0.05, *P < 0.05, **P < 0.01, ***P < 0.001.
(TIF)

S3 Fig Overexpression of C3AR1 or ITGB2 promotes oxidative stress, inflammation and apoptosis in HK-2 cells. HK-2 cells were transfected with pcDNA-C3AR1 (1.0 μg/mL) or pcDNA-ITGB2 (1.0 μg/mL) for 48 hours. (A) HK-2 cell viability was detected by MTT assay; (B) MDA content detection kit to detect MDA content; (C) NADPH assay kit was used to analyze NADPH activity; (D) GSH detection kit to detect GSH concentration; (E) SOD detection kit to detect SOD activity; (F) QPCR was used to detect the expression of inflammatory factor TNF-α mRNA in HK-2 cells; (G) QPCR was used to detect the expression of inflammatory factor IL-6 mRNA in HK-2 cells; (H) QPCR was used to detect the expression level of inflammatory factor IL-1β mRNA in HK-2 cells; (I) ELISA was used to detect the secretion level of inflammatory factor TNF-α in HK-2 cell culture supernatant; (J) ELISA was used to detect the secretion level of inflammatory factor IL-6 in HK-2 cell culture supernatant; (K) ELISA was used to detect the secretion level of inflammatory factor IL-1β in HK-2 cell culture supernatant; (L) Flow cytometry was used to detect the level of apoptosis. Data shown are the mean ± SD, N = 4. The statistical differences were evaluated by one-way ANOVA, and followed by LSD test. Compared with the control group, the pcDNA-vector group, ns *P* > 0.05, *P < 0.05, **P < 0.01.
(TIF)

S1 File Raw images. Original images for blot and gel.
(PDF)

## Abbreviations

DN: Diabetes nephropathy
HG: high glucose
HK-2 cells: Human renal tubular epithelial cells
Co-IP: Co-Immunoprecipitation analysis
STZ: treptozotocin
ESRD: End-stage renal disease
C3AR1: C3a anaphylatoxin chemotactic receptor
ITGB2: Integrin subunit beta 2
QPCR: Quantitative real-time reverse transcription PCR
ROS: Reactive Oxygen Species
MDA: Malondialdehyde
NADPH: Nicotinamide Adenine Dinucleotide Phosphate Oxidase
GSH: Glutathione
SOD: Superoxide Dismutase
BUN: Blood urea nitrogen
Cr: Creatinine
TNF-α: Tumour Necrosis Factor alpha
IL-6: Interleukin 6
IL-1β: Interleukin-1beta
CHX: Cycloheximide
DCFH-DA: 2’,7’-Dichlorodihydrofluorescein diacetate
