# Supplementary figures and images for: C3AR1 may aggravate diabetic nephropathy by mediating oxidative stress via ITGB2 regulation in renal tubular epithelial cells
Source: PLoS One. 2025 Sep 12;20(9):e0331900. doi: 10.1371/journal.pone.0331900 (PMC12431250; doi:10.1371/journal.pone.0331900)

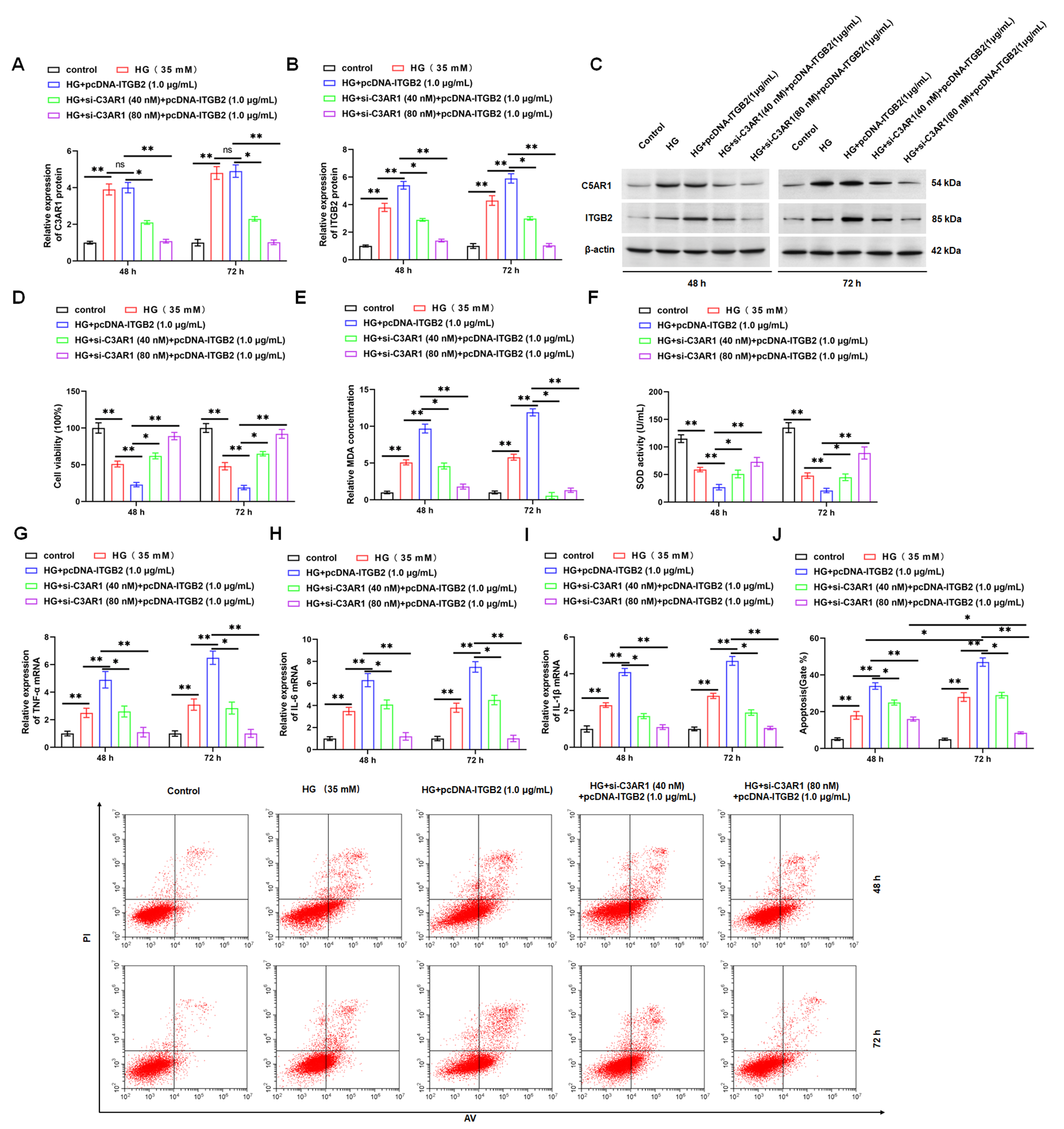

Supplement: S1 Fig — HK-2 cells were treated with 5.5 mM glucose as control, and HK-2 cells were treated with 35 mM HG for 24 hours to establish a DN cell model, and then transfected with C3AR1 siRNA (40 and 80 nM) or/and pcDNA-ITGB2 (0.5 and 1.0 μg/mL) for 48 and 72 hours. (A-C) Western blotting was used to detect the expression level of C3AR1 protein in HK-2 cells; (D) HK-2 cell viability was detected by MTT assay; (E) MDA content detection kit to detect MDA content; (F) SOD detection kit to detect SOD activity; (G) QPCR was used to detect the expression of inflammatory factor TNF-α mRNA in HK-2 cells; (H) QPCR was used to detect the expression of inflammatory factor IL-6 mRNA in HK-2 cells; (I) QPCR was used to detect the expression level of inflammatory factor IL-1β mRNA in HK-2 cells; (J) Flow cytometry was used to detect the level of apoptosis. Data shown are the mean ± SD, N = 4. The statistical differences were evaluated by one-way or two-way ANOVA, and followed by LSD test. Compared with the control group, the HG group, the HG + pcDNA-ITGB2 (1.0 μg/mL) group, ns P > 0.05, *P < 0.05, **P < 0.01. (TIF) [file pone.0331900.s001.tif]

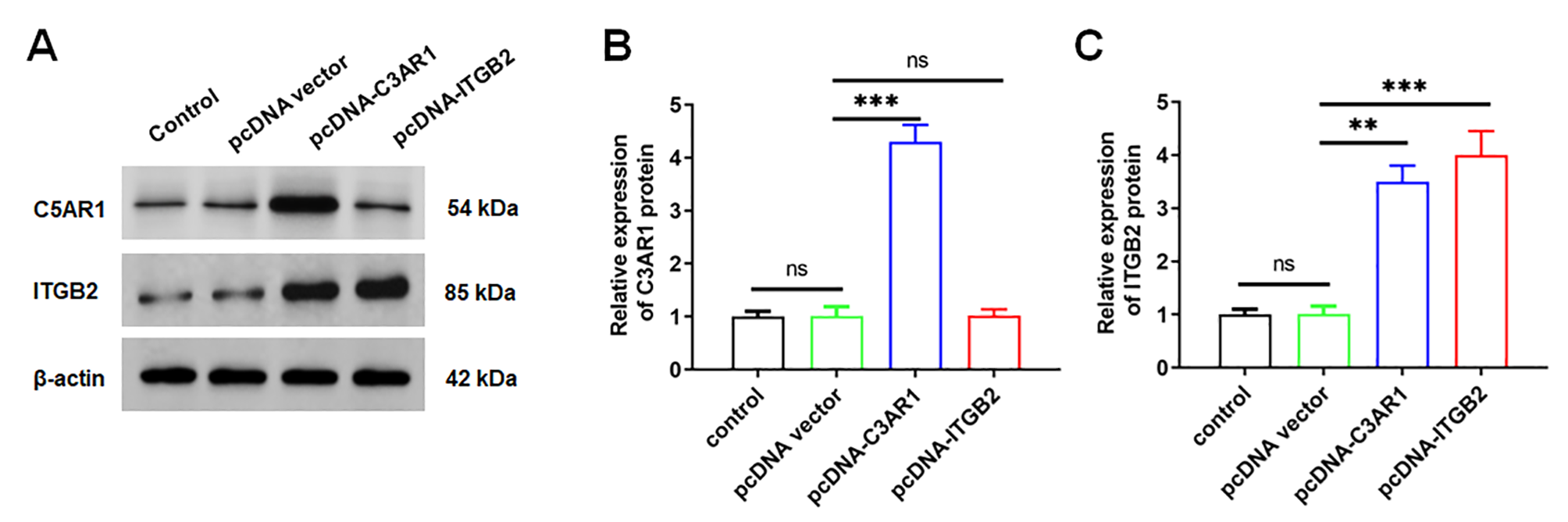

Supplement: S2 Fig — HK-2 cells were transfected with pcDNA-C3AR1 (1.0 μg/mL) or pcDNA-ITGB2 (1.0 μg/mL) for 48 hours. (A) Western blotting was used to detect the protein levels of C3AR1 (B) and ITGB2 (C). Data shown are the mean ± SD, N = 4. The statistical differences were evaluated by one-way ANOVA, and followed by LSD test. Compared with the control group, the pcDNA-vector group, ns P > 0.05, *P < 0.05, **P < 0.01, ***P < 0.001. (TIF) [file pone.0331900.s002.tif]

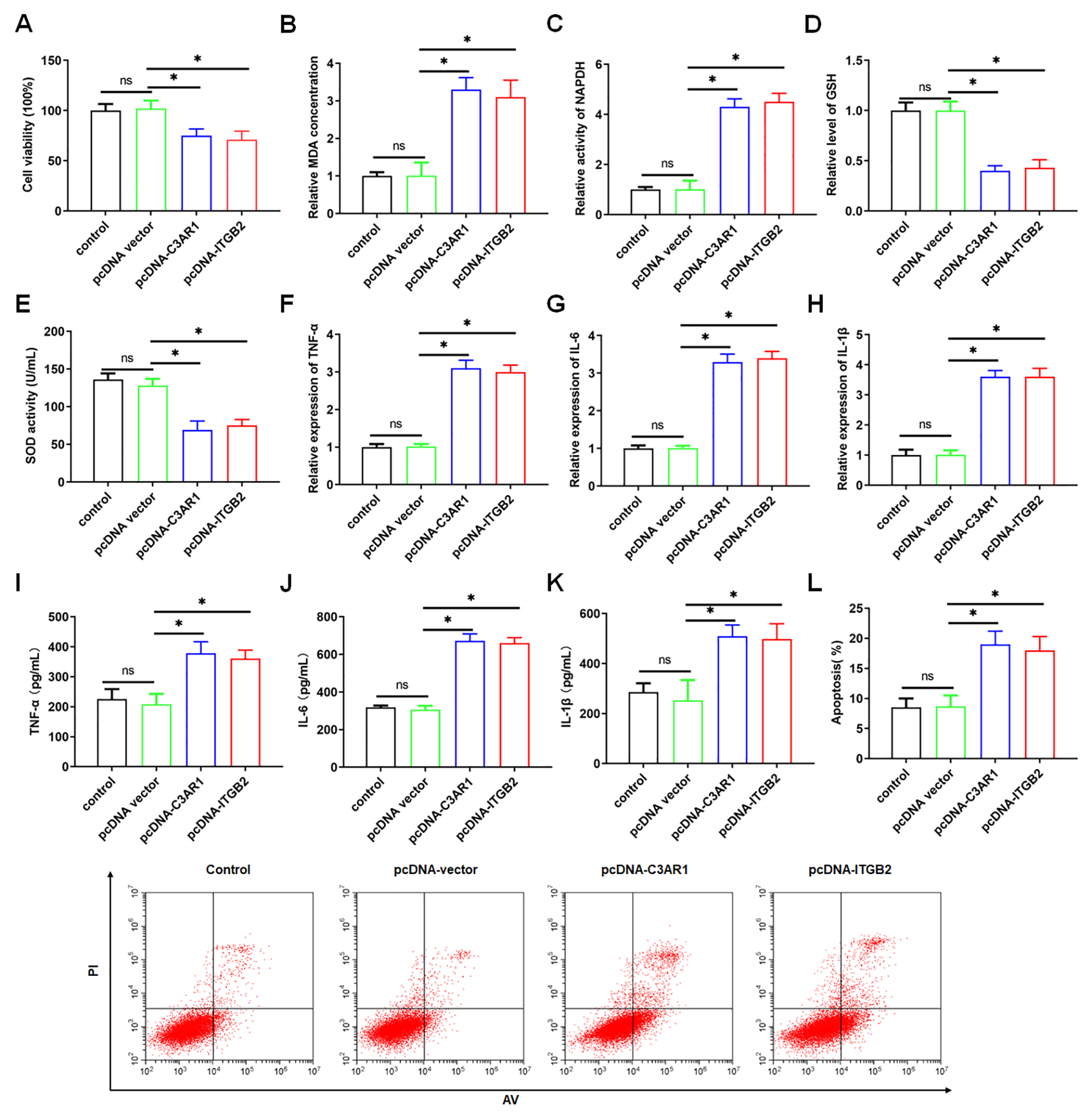

Supplement: S3 Fig — HK-2 cells were transfected with pcDNA-C3AR1 (1.0 μg/mL) or pcDNA-ITGB2 (1.0 μg/mL) for 48 hours. (A) HK-2 cell viability was detected by MTT assay; (B) MDA content detection kit to detect MDA content; (C) NADPH assay kit was used to analyze NADPH activity; (D) GSH detection kit to detect GSH concentration; (E) SOD detection kit to detect SOD activity; (F) QPCR was used to detect the expression of inflammatory factor TNF-α mRNA in HK-2 cells; (G) QPCR was used to detect the expression of inflammatory factor IL-6 mRNA in HK-2 cells; (H) QPCR was used to detect the expression level of inflammatory factor IL-1β mRNA in HK-2 cells; (I) ELISA was used to detect the secretion level of inflammatory factor TNF-α in HK-2 cell culture supernatant; (J) ELISA was used to detect the secretion level of inflammatory factor IL-6 in HK-2 cell culture supernatant; (K) ELISA was used to detect the secretion level of inflammatory factor IL-1β in HK-2 cell culture supernatant; (L) Flow cytometry was used to detect the level of apoptosis. Data shown are the mean ± SD, N = 4. The statistical differences were evaluated by one-way ANOVA, and followed by LSD test. Compared with the control group, the pcDNA-vector group, ns P > 0.05, *P < 0.05, **P < 0.01. (TIF) [file pone.0331900.s003.tif]
