# Supplementary material for: C3AR1 may aggravate diabetic nephropathy by mediating oxidative stress via ITGB2 regulation in renal tubular epithelial cells
Source: PLoS One. 2025 Sep 12;20(9):e0331900. doi: 10.1371/journal.pone.0331900 (PMC12431250; doi:10.1371/journal.pone.0331900)

Fig.1B-C3AR1

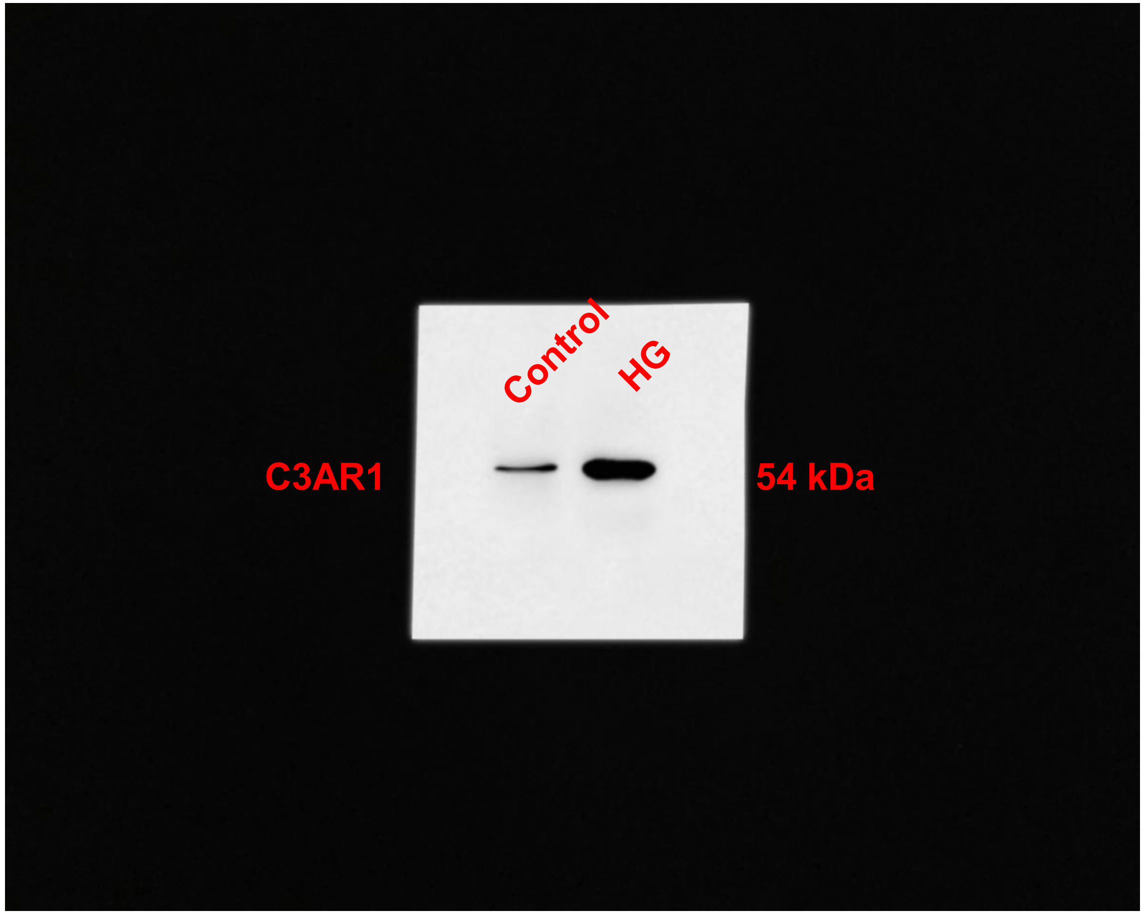

Fig.1B-β-actin

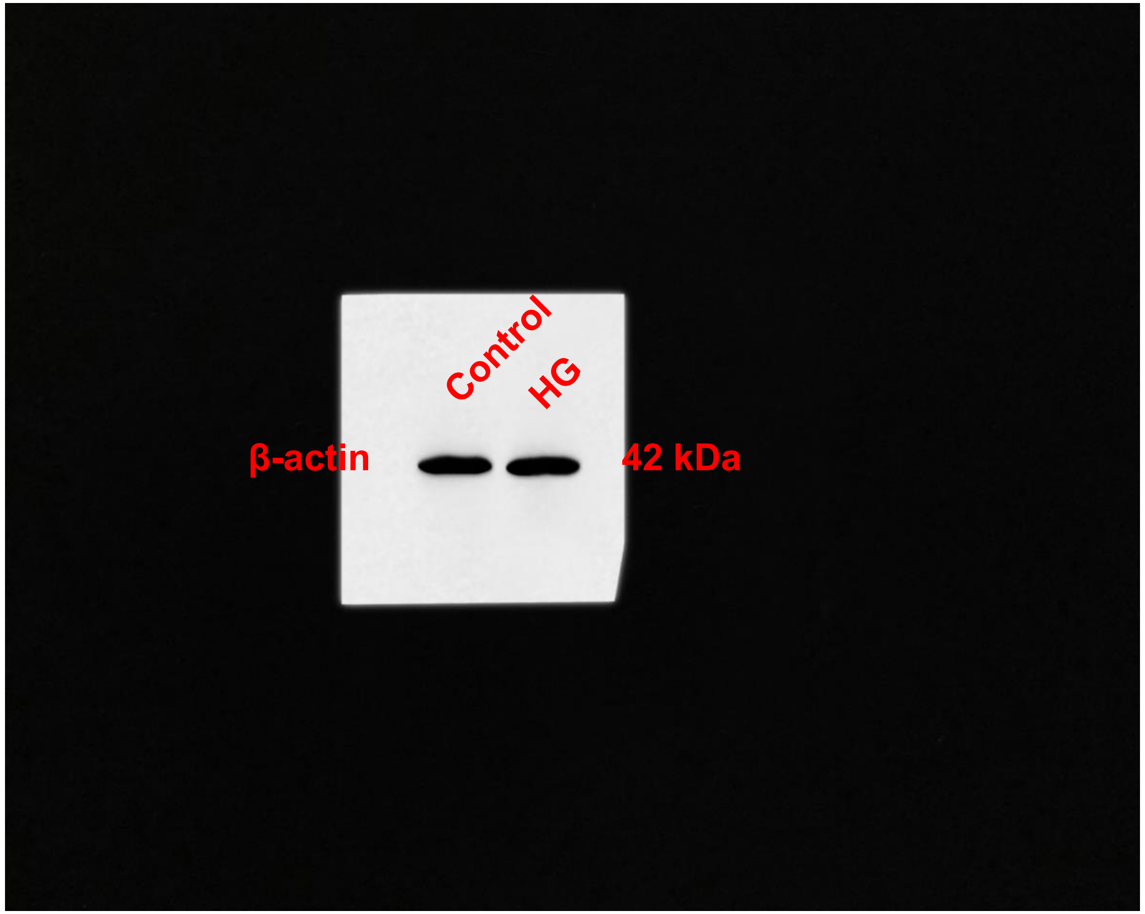

Fig.2B-C3AR1

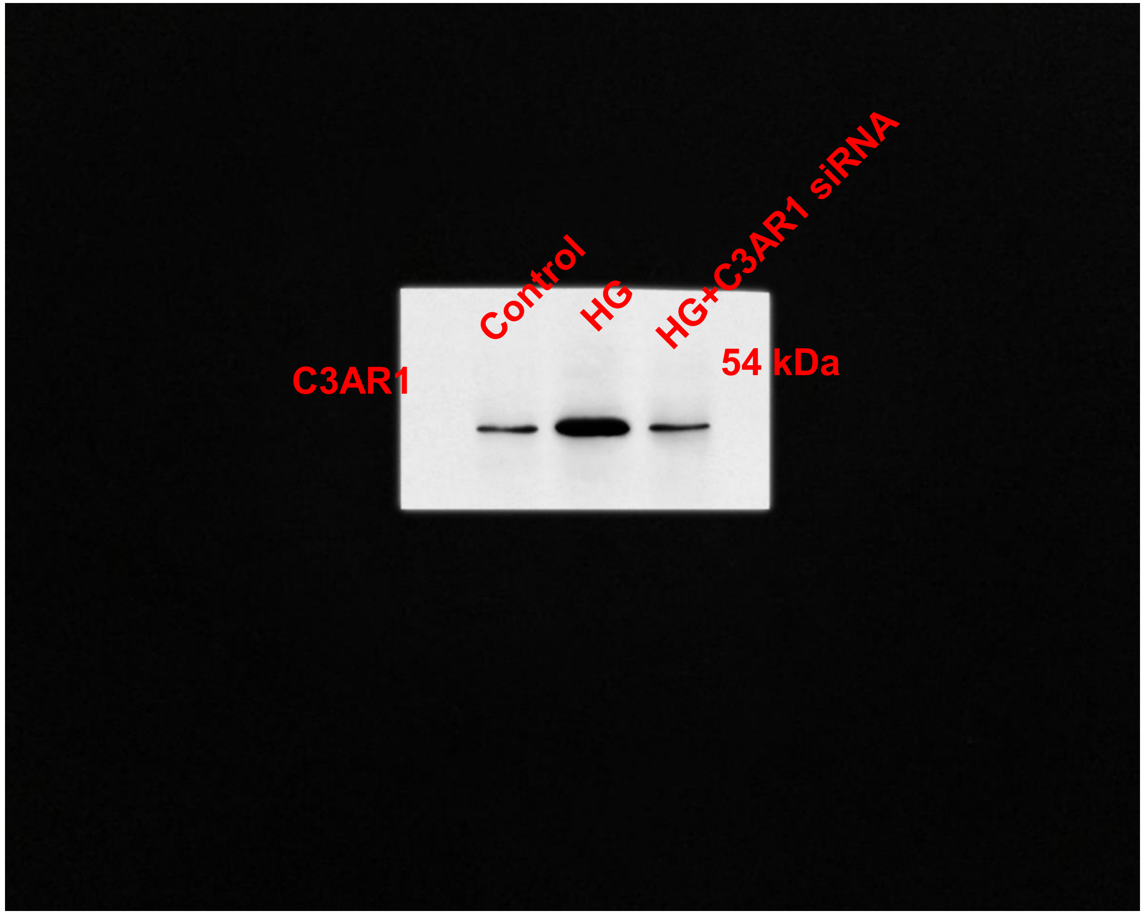

Fig.2B-β-actin

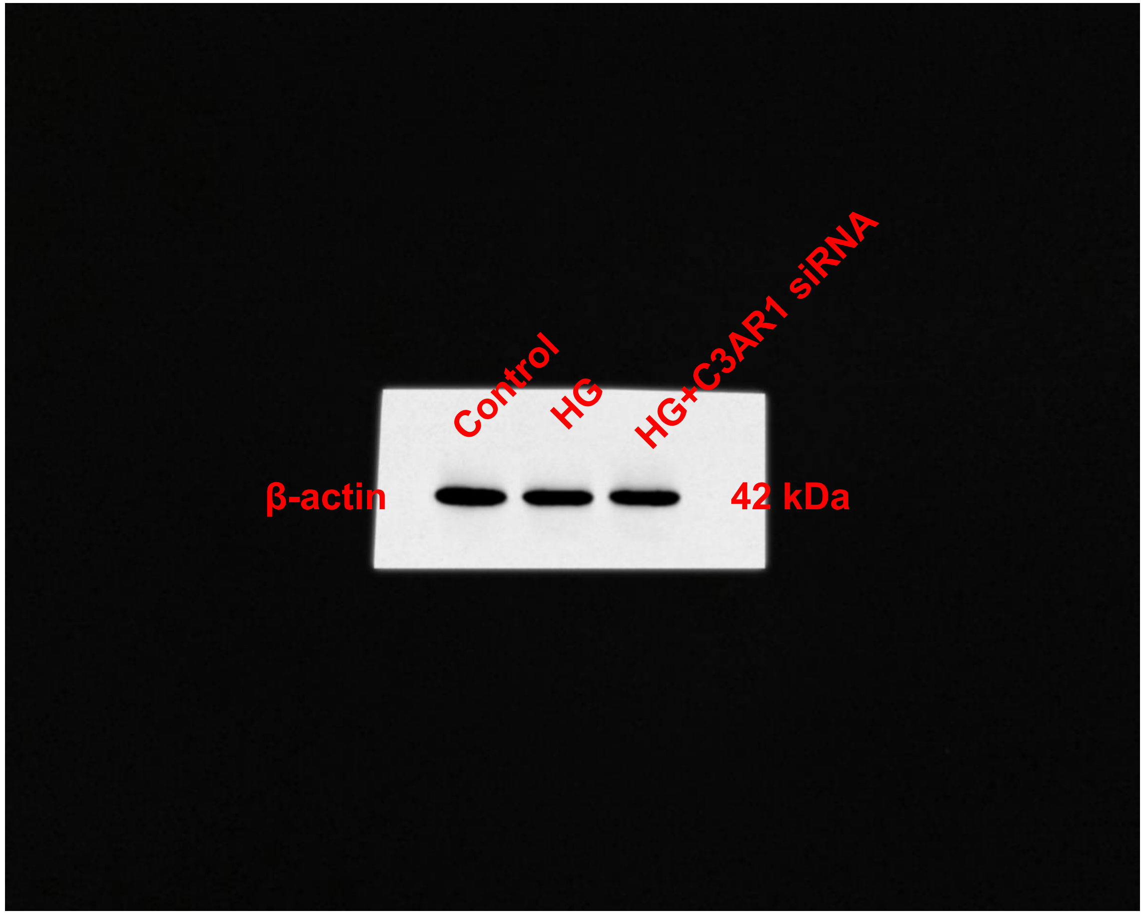

Fig.4C-ITGB2

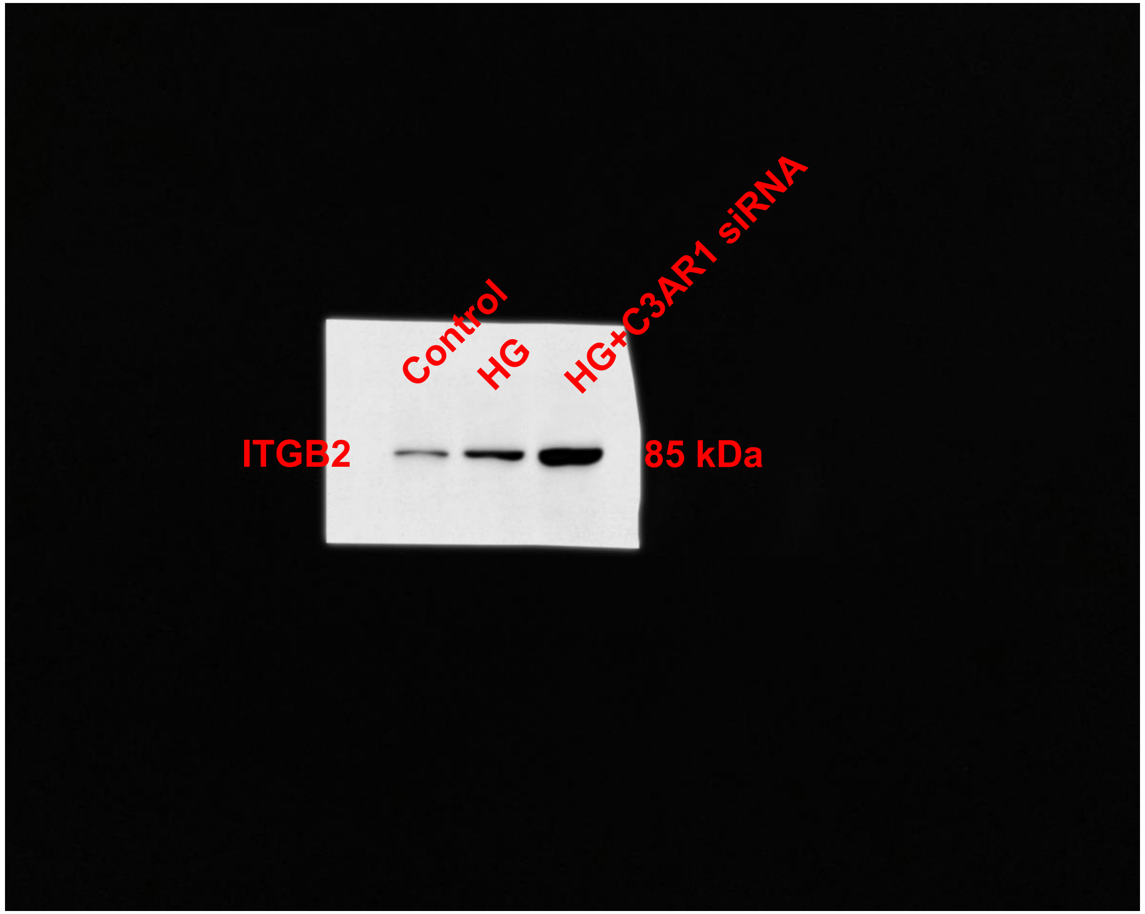

Fig.4C-β-actin

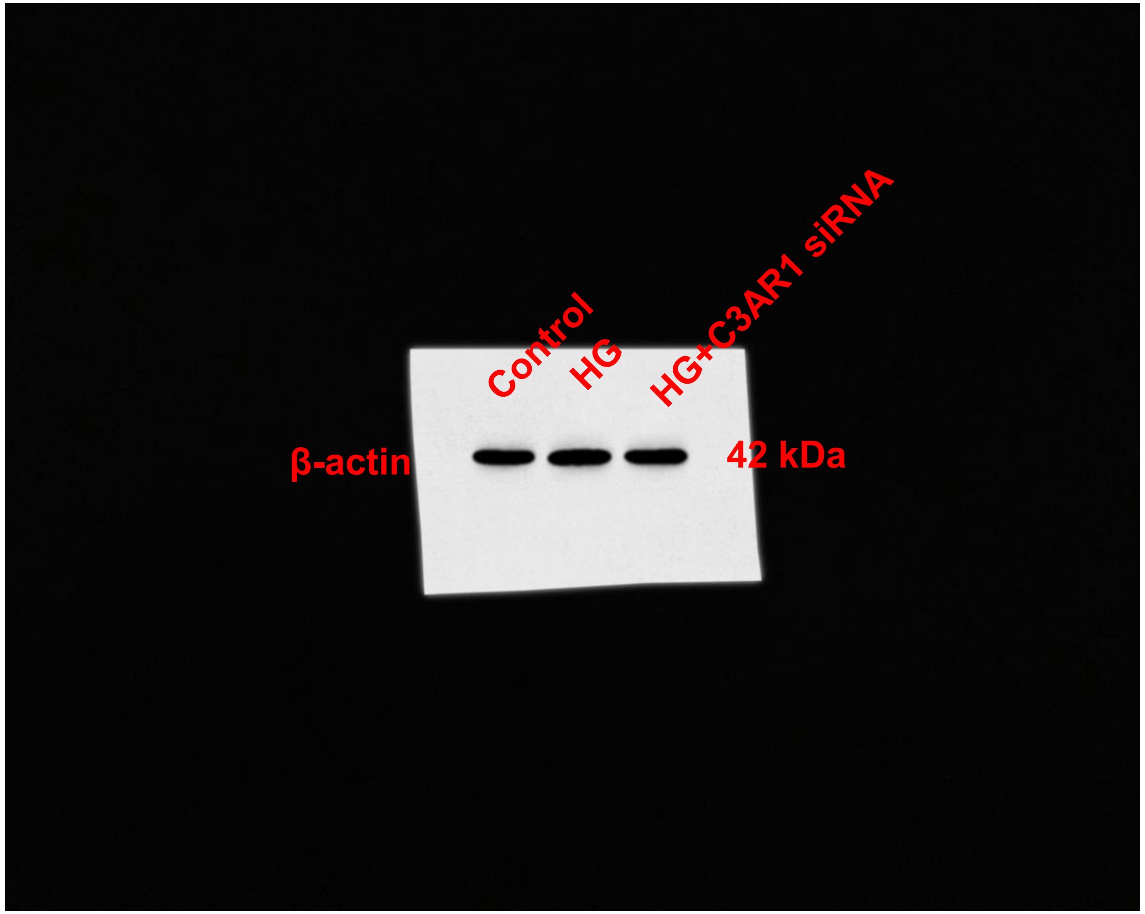

Fig.4E-ITGB2

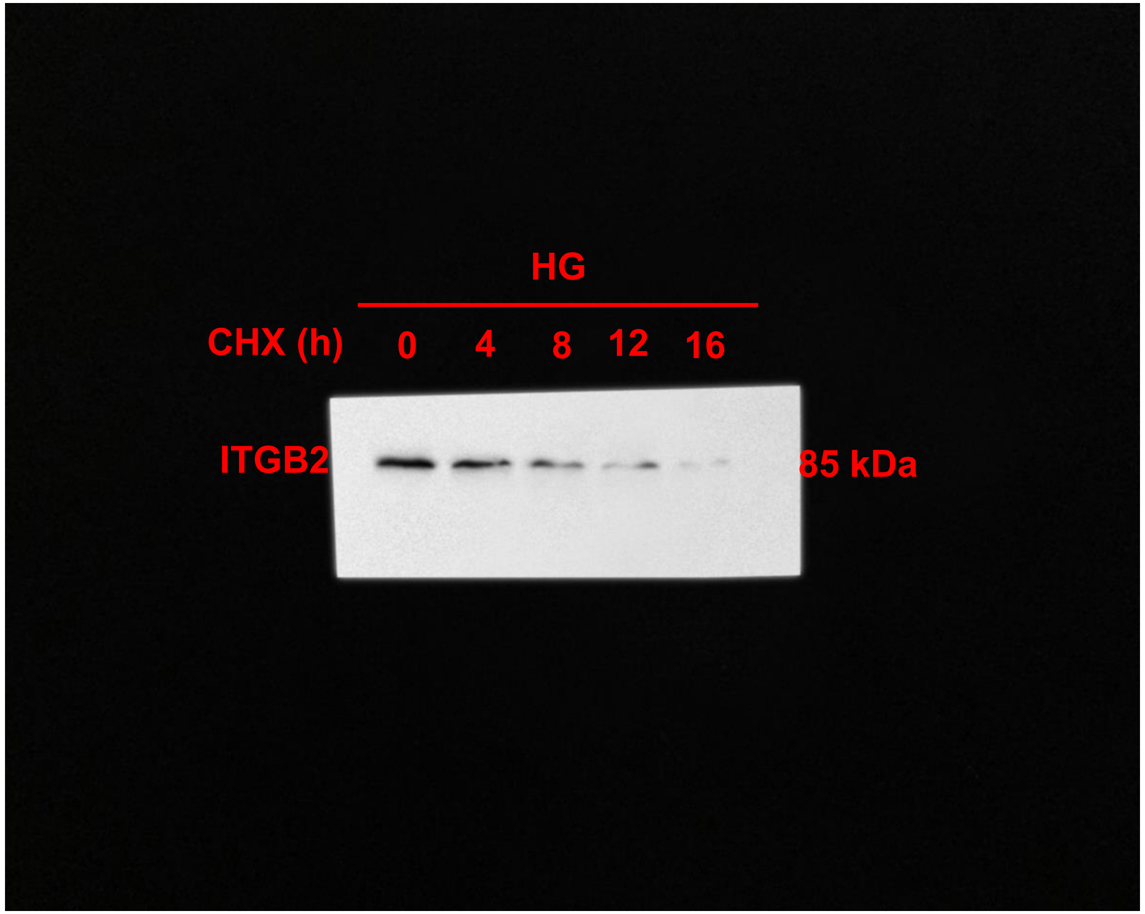

Fig.4E-β-actin

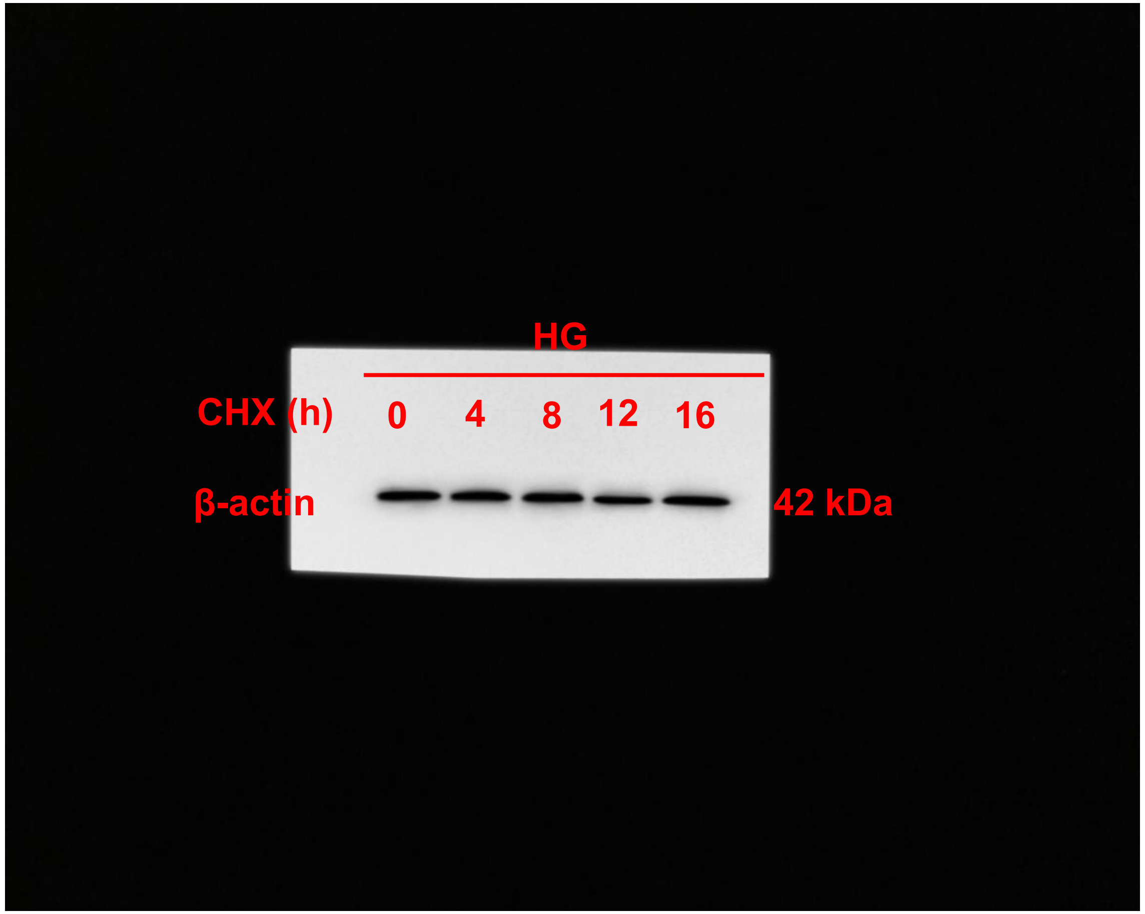

Fig.4E-ITGB2

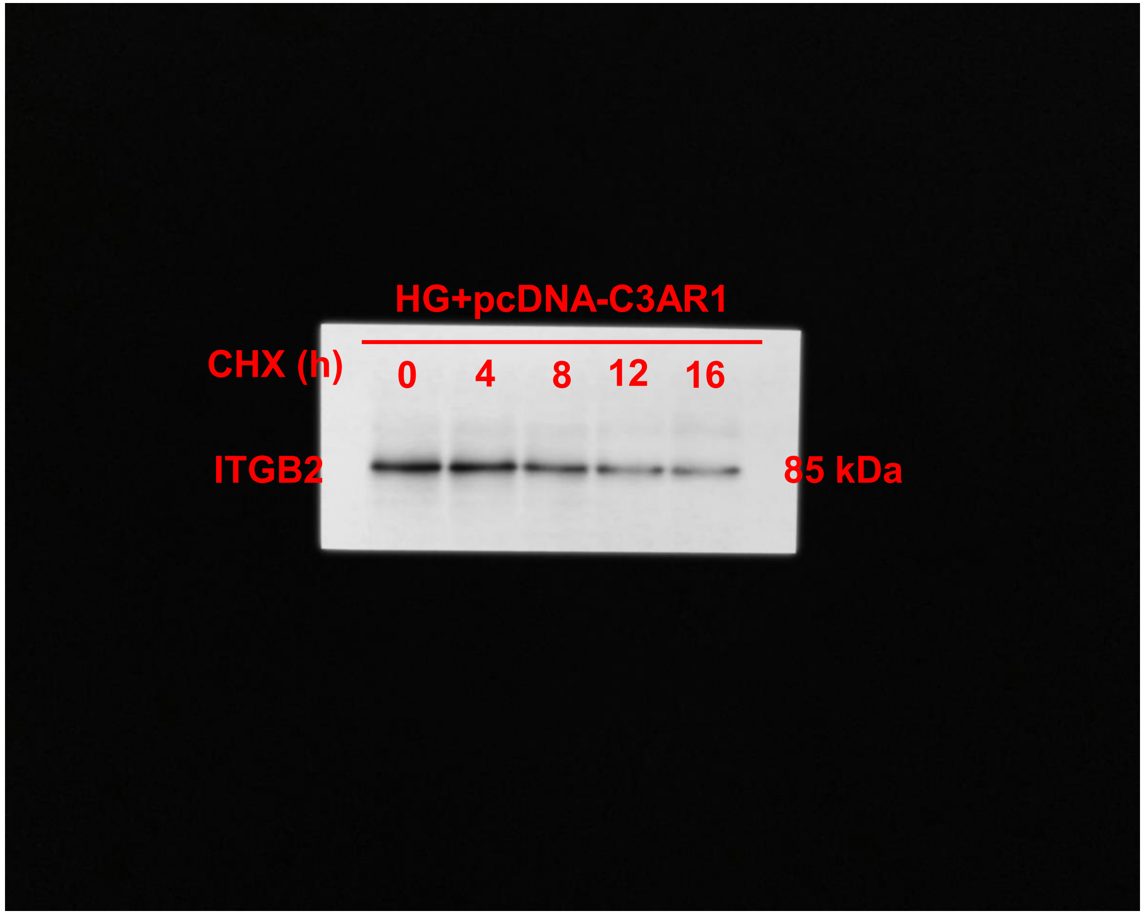

Fig.4E-β-actin

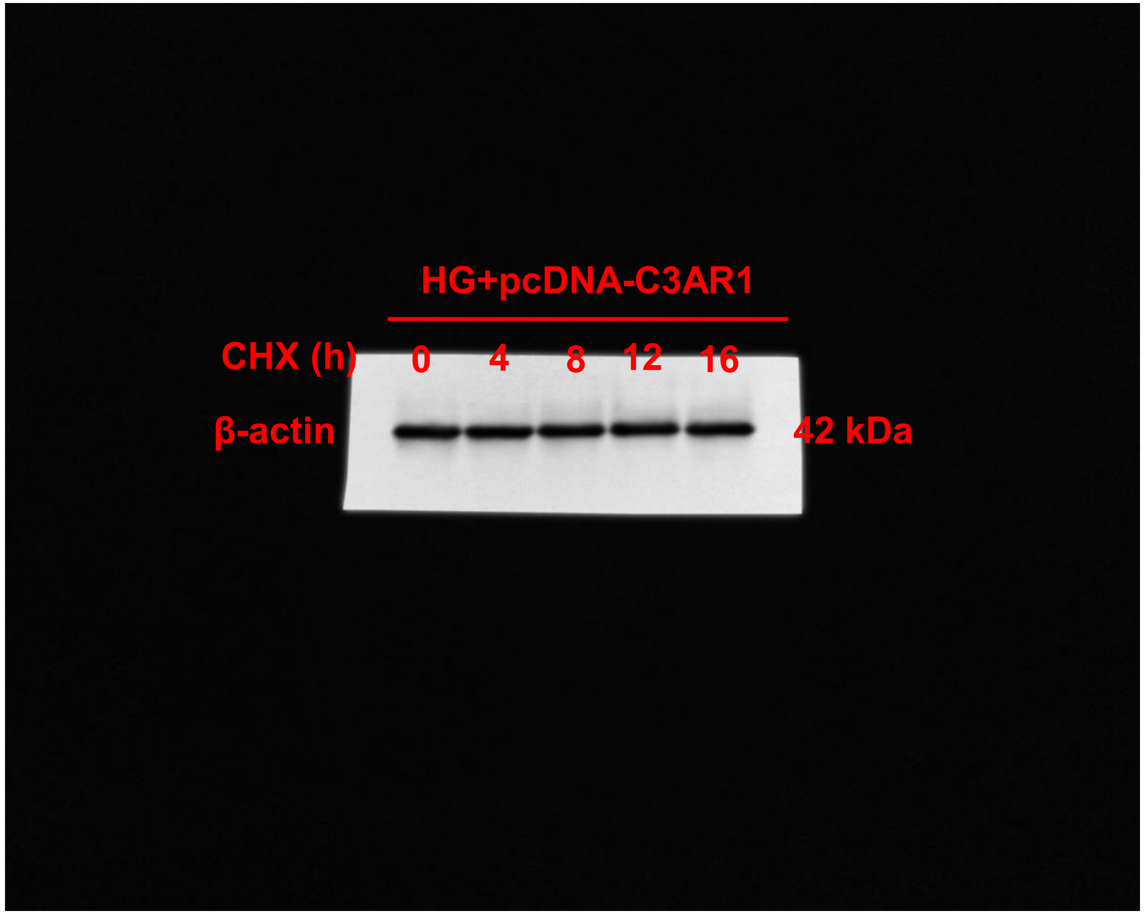

Fig.5B-ITGB2

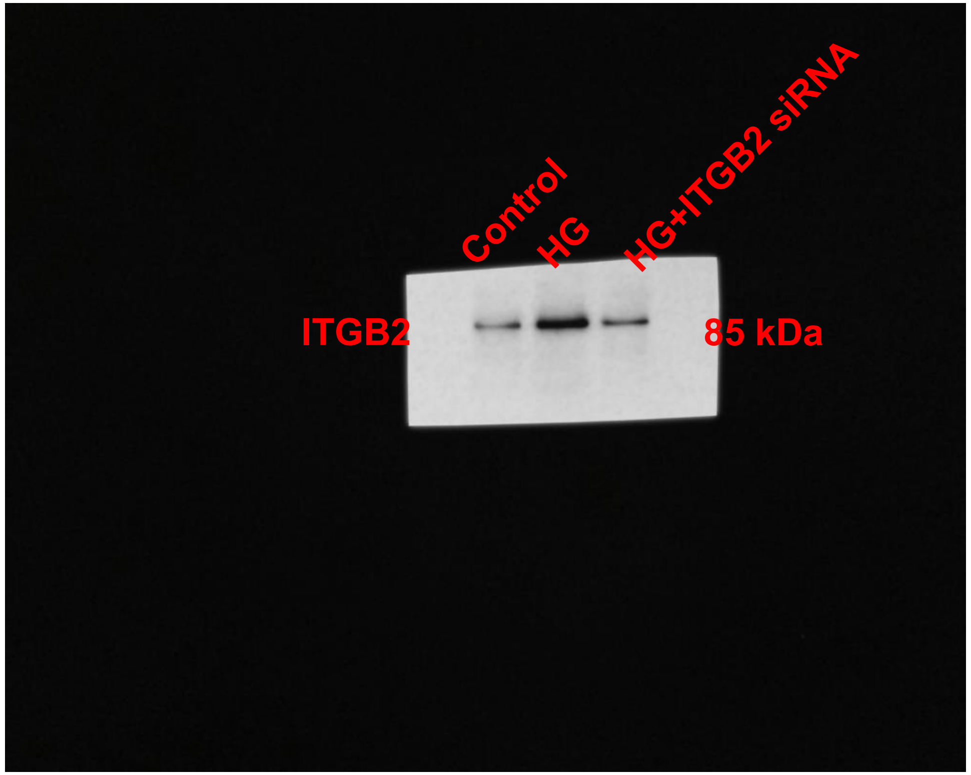

Fig.5B-β-actin

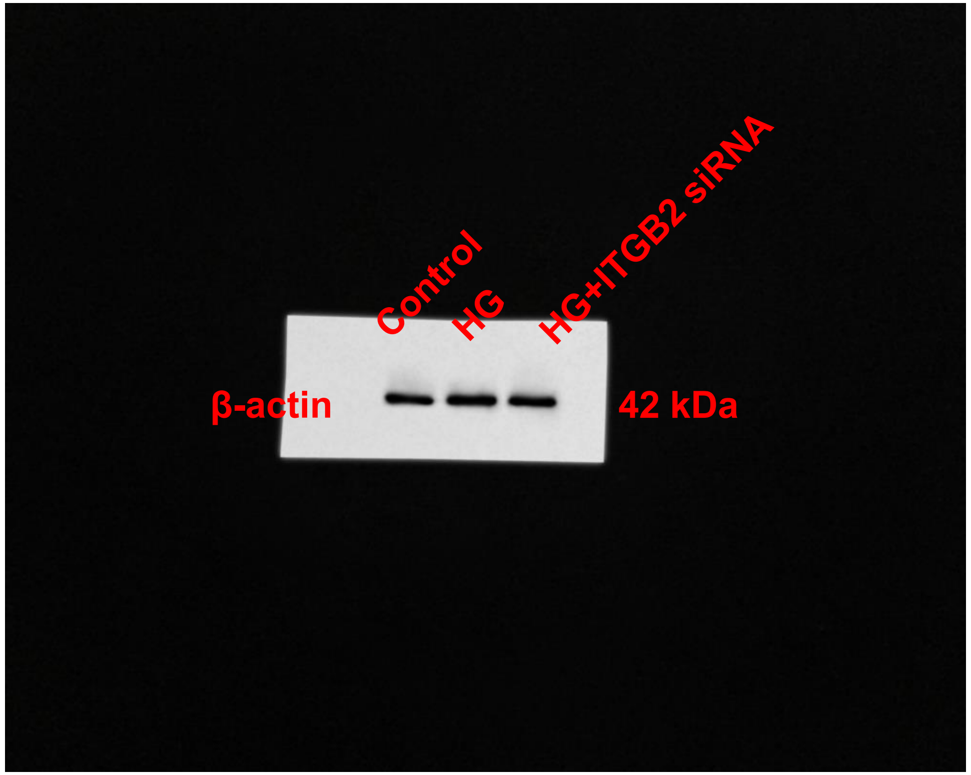

Fig.7A-C3AR1

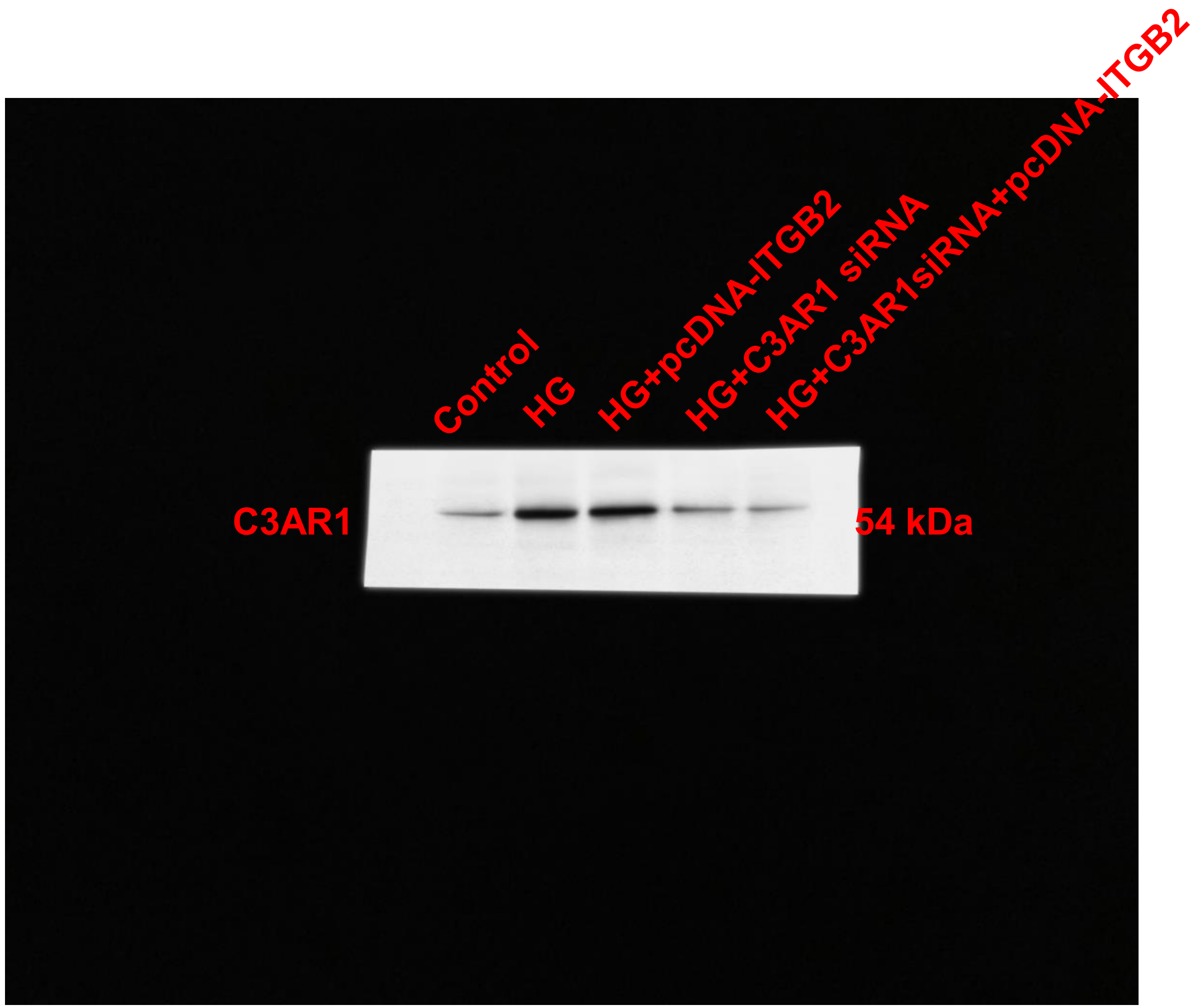
Control
HG
HG+pcDNA-ITGB2
HG+C3AR1 siRNA
HG+C3AR1siRNA+pcDNA-ITGB2
C3AR1
54 kDa

Fig.7A-ITGB2

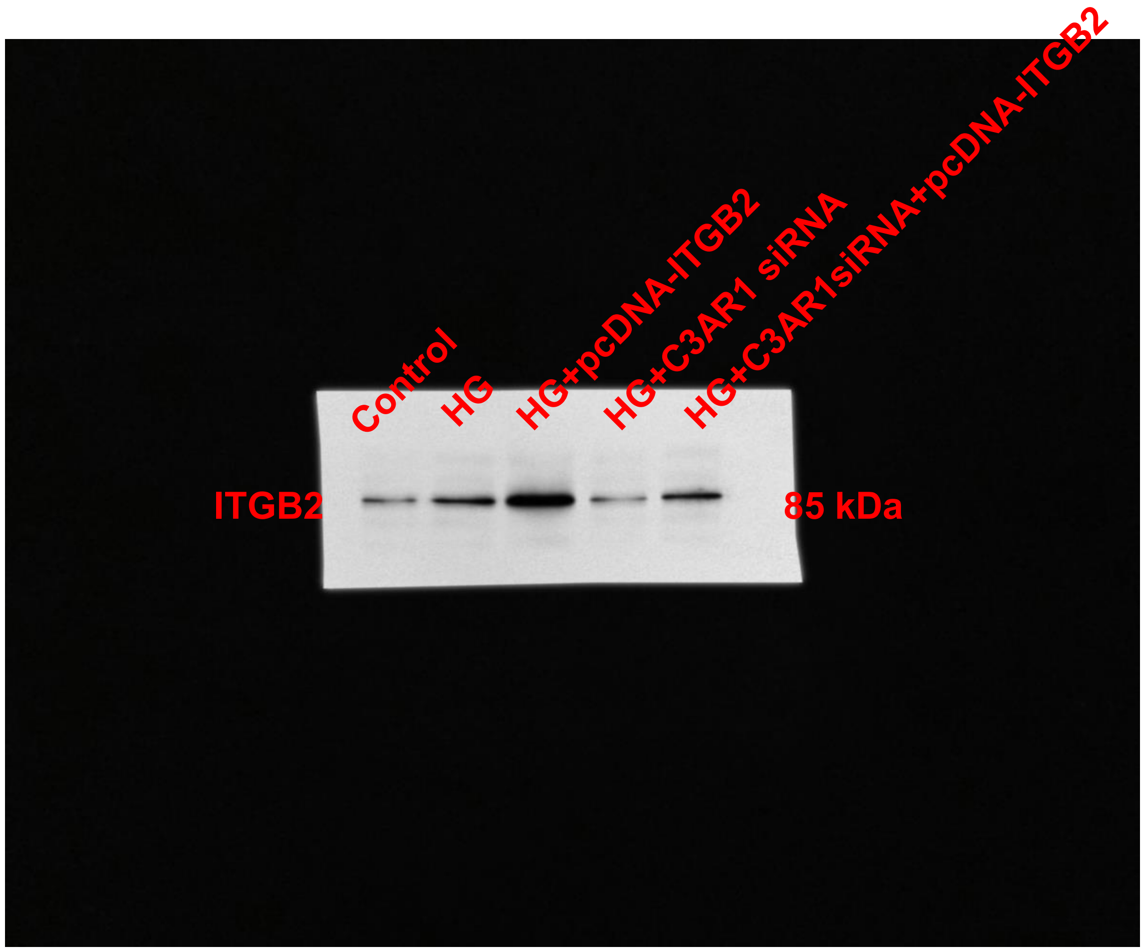
Control
HG
HG+pcDNA-ITGB2
HG+C3AR1 siRNA
HG+C3AR1siRNA+pcDNA-ITGB2
ITGB2
85 kDa

Fig.7A-β-actin

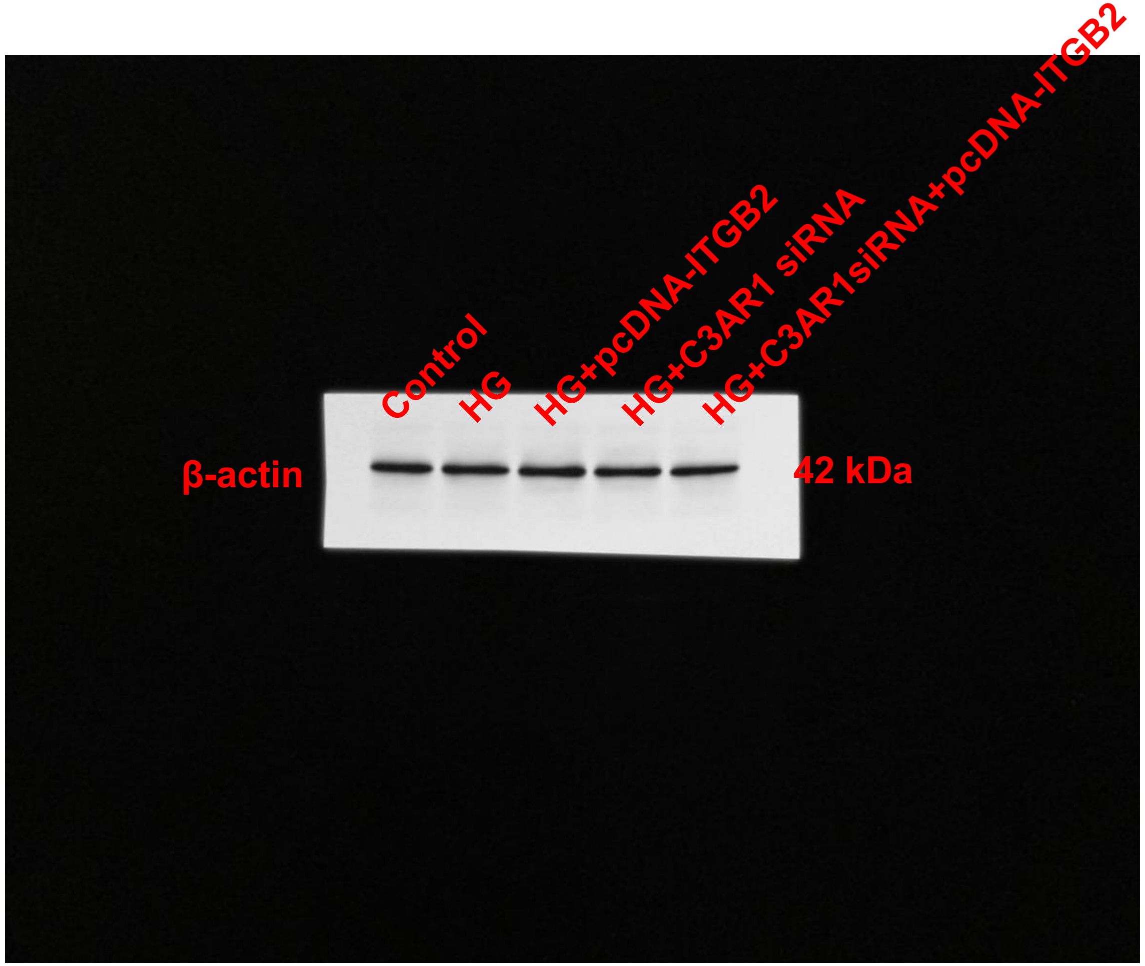

Fig.9A-C3AR1

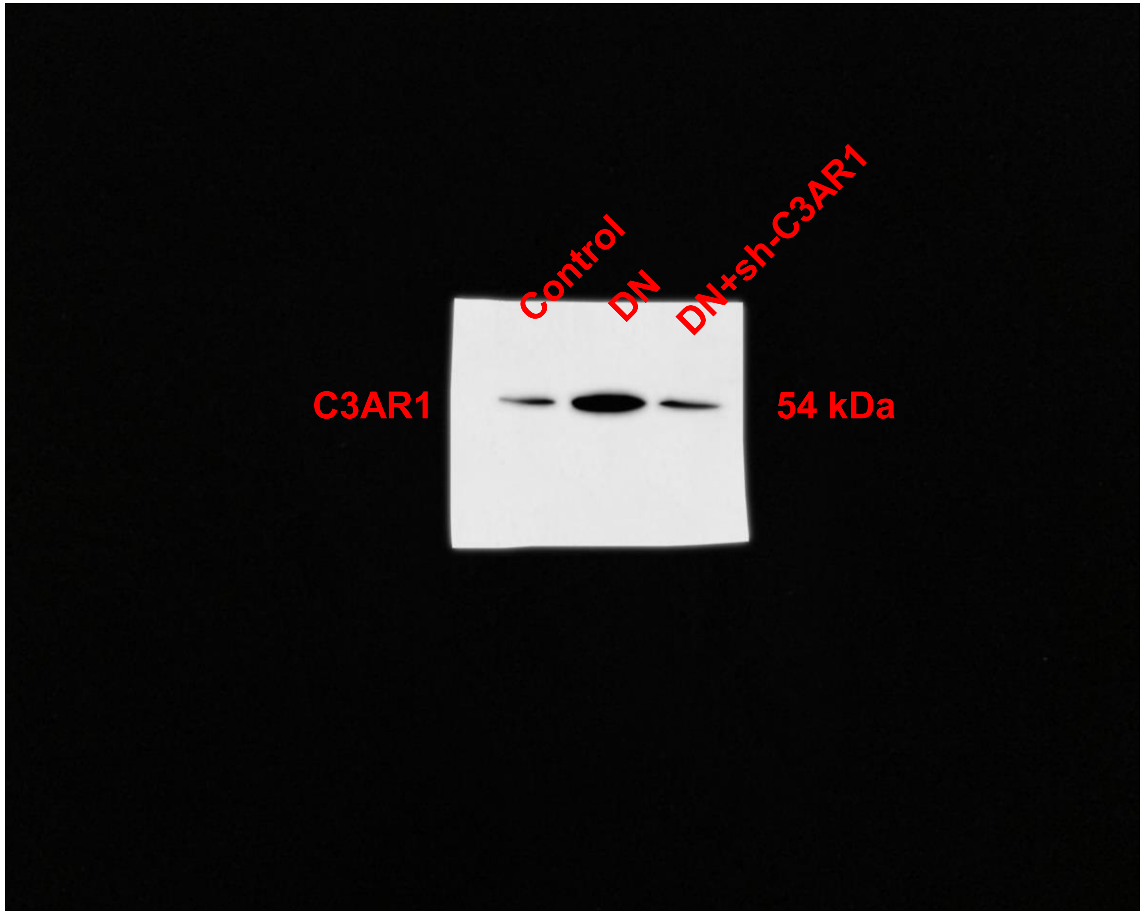

Fig.9A-ITGB2

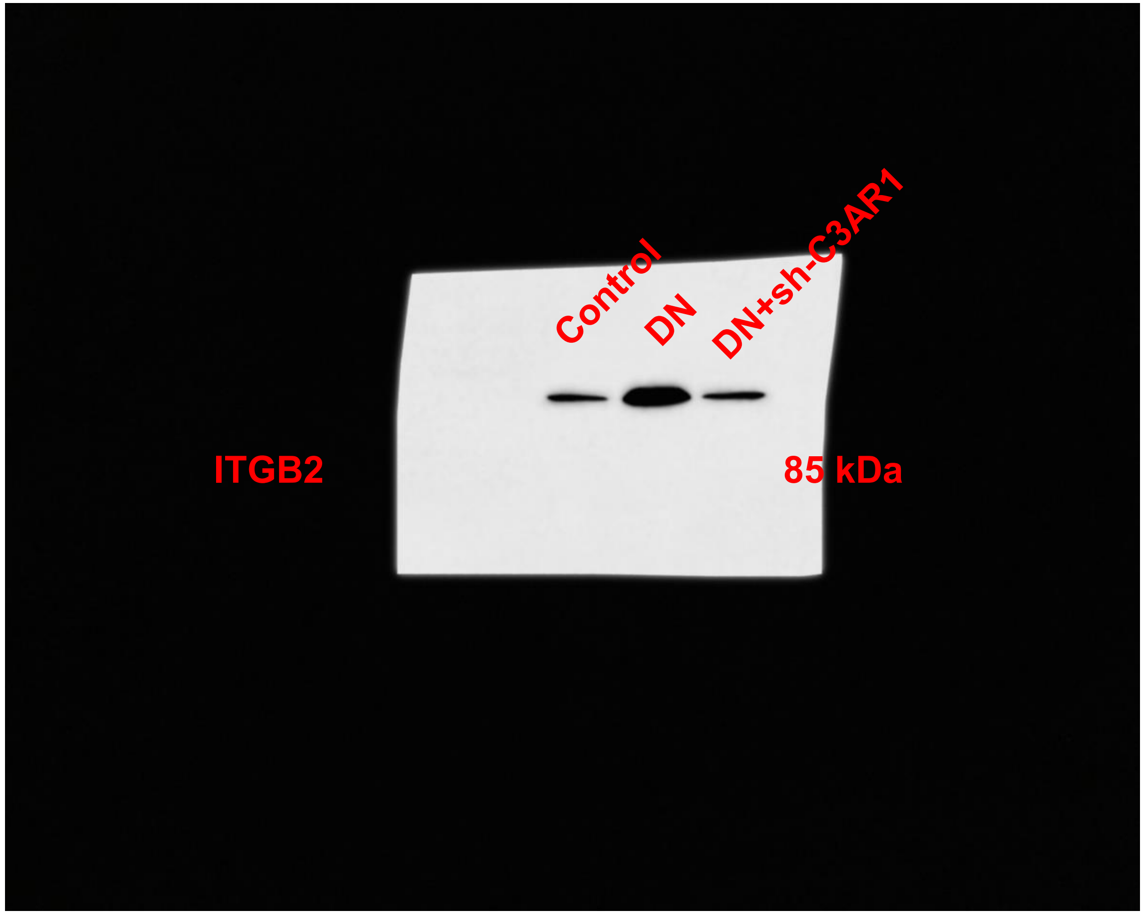

Fig.9A-β-actin

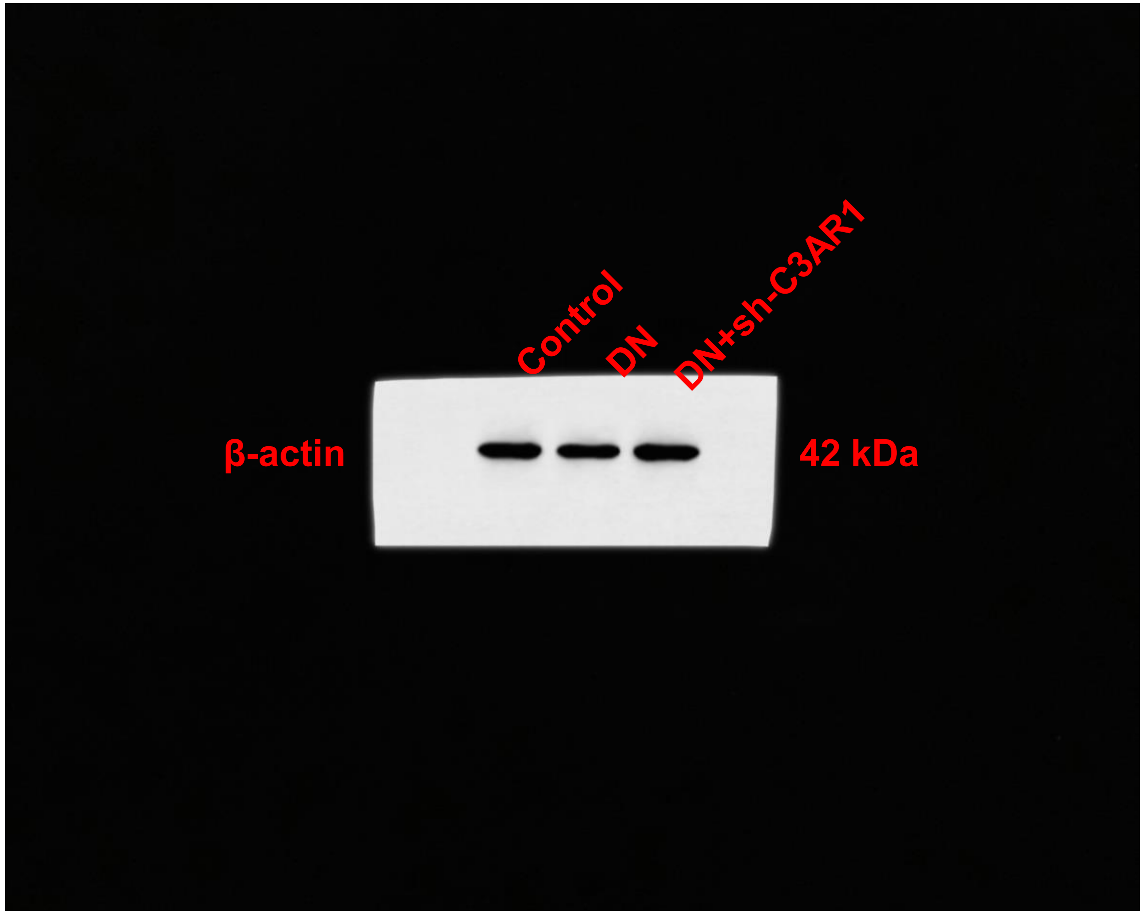

S-Fig.1C-C3AR1

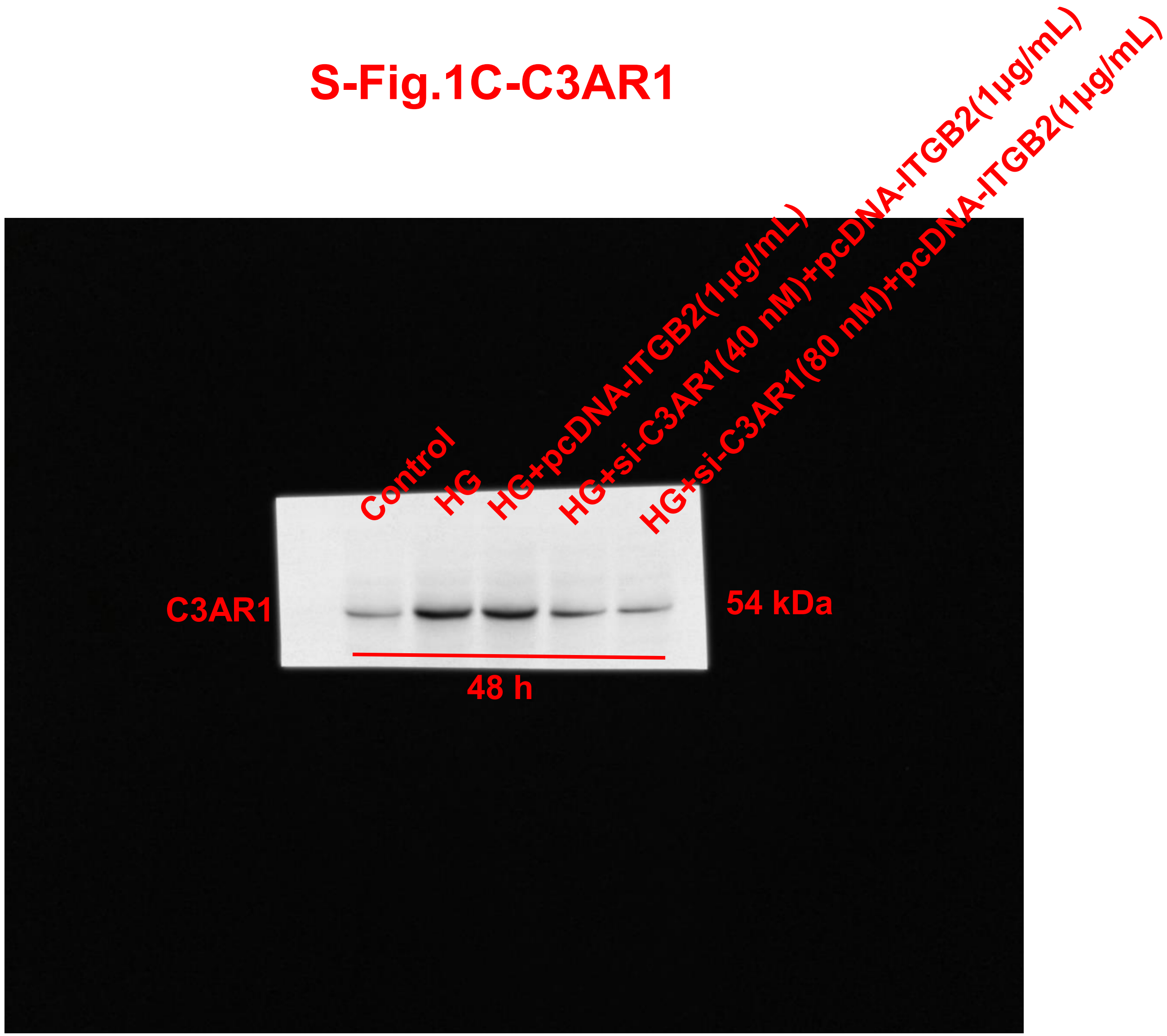

S-Fig.1C-ITGB2

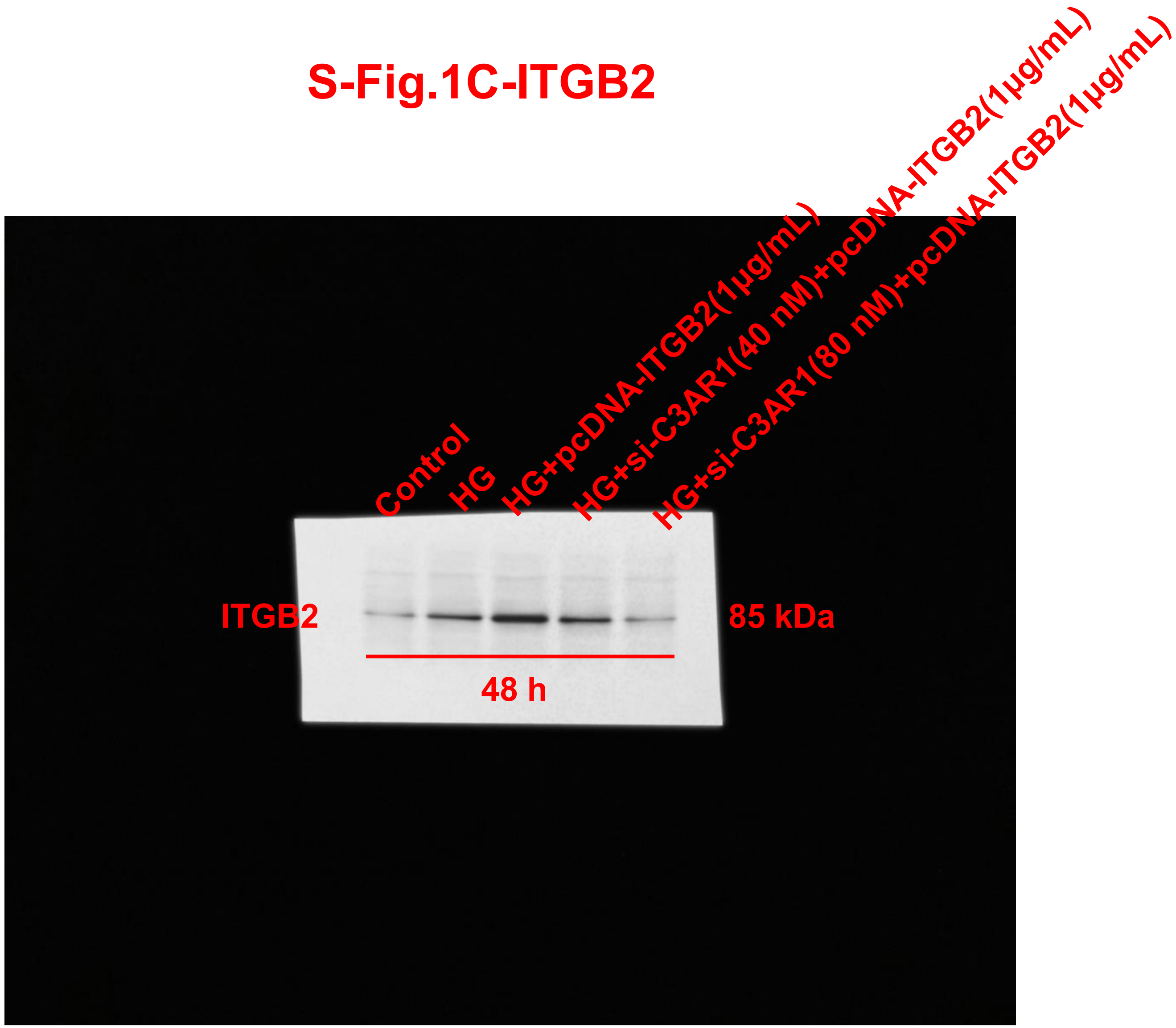

S-Fig.1C-β-actin

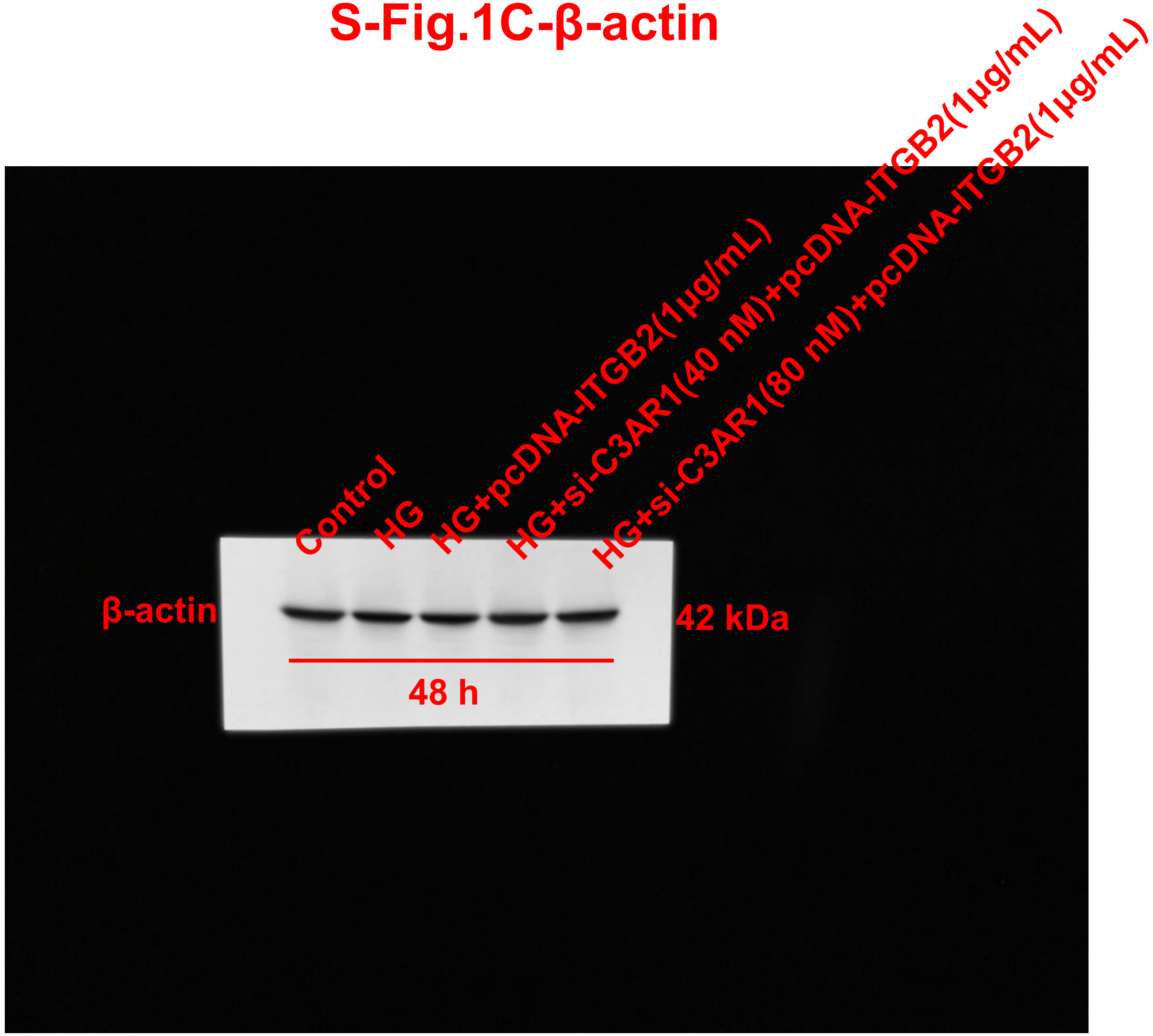

S-Fig.1C-C3AR1

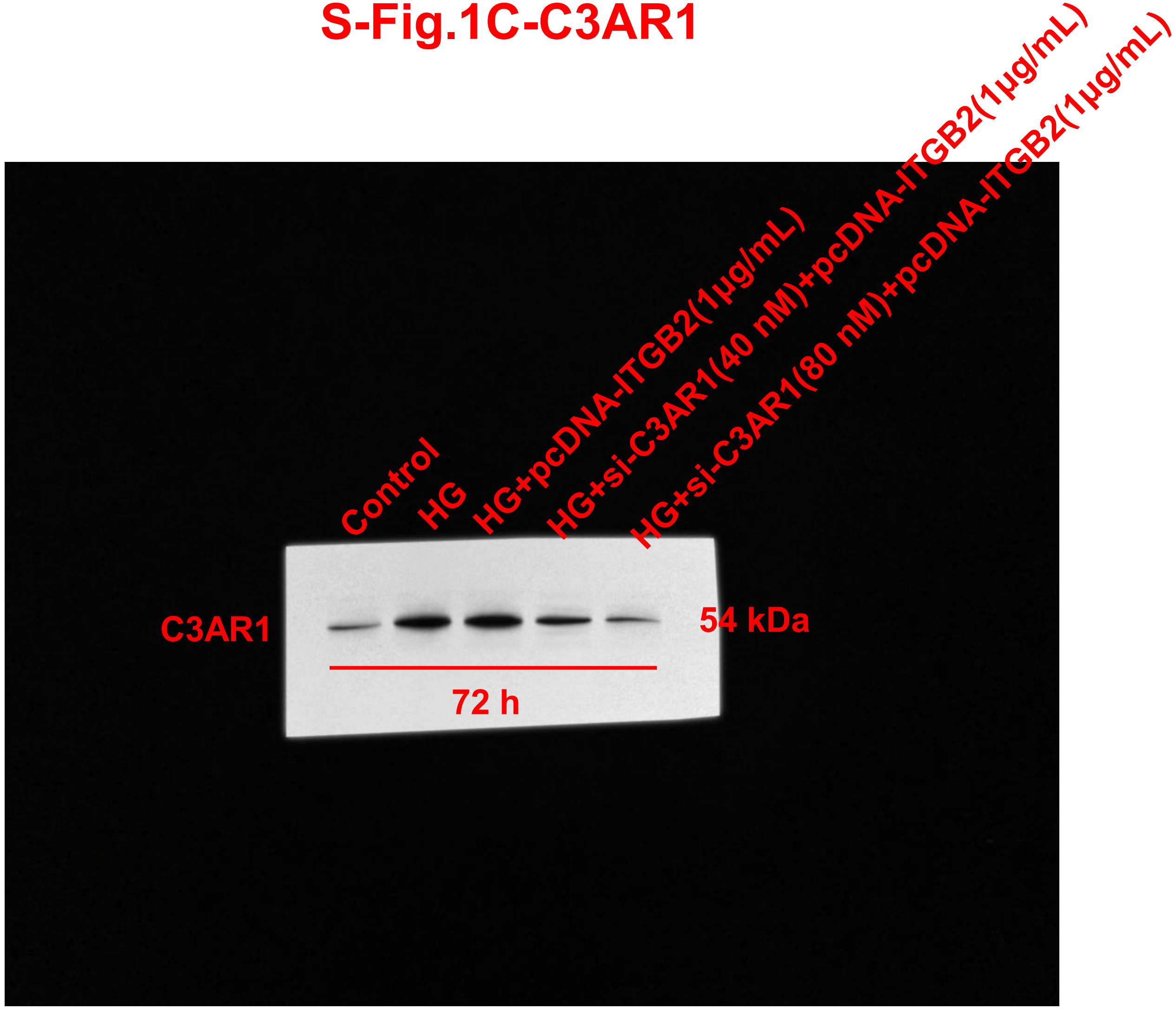

S-Fig.1C-ITGB2

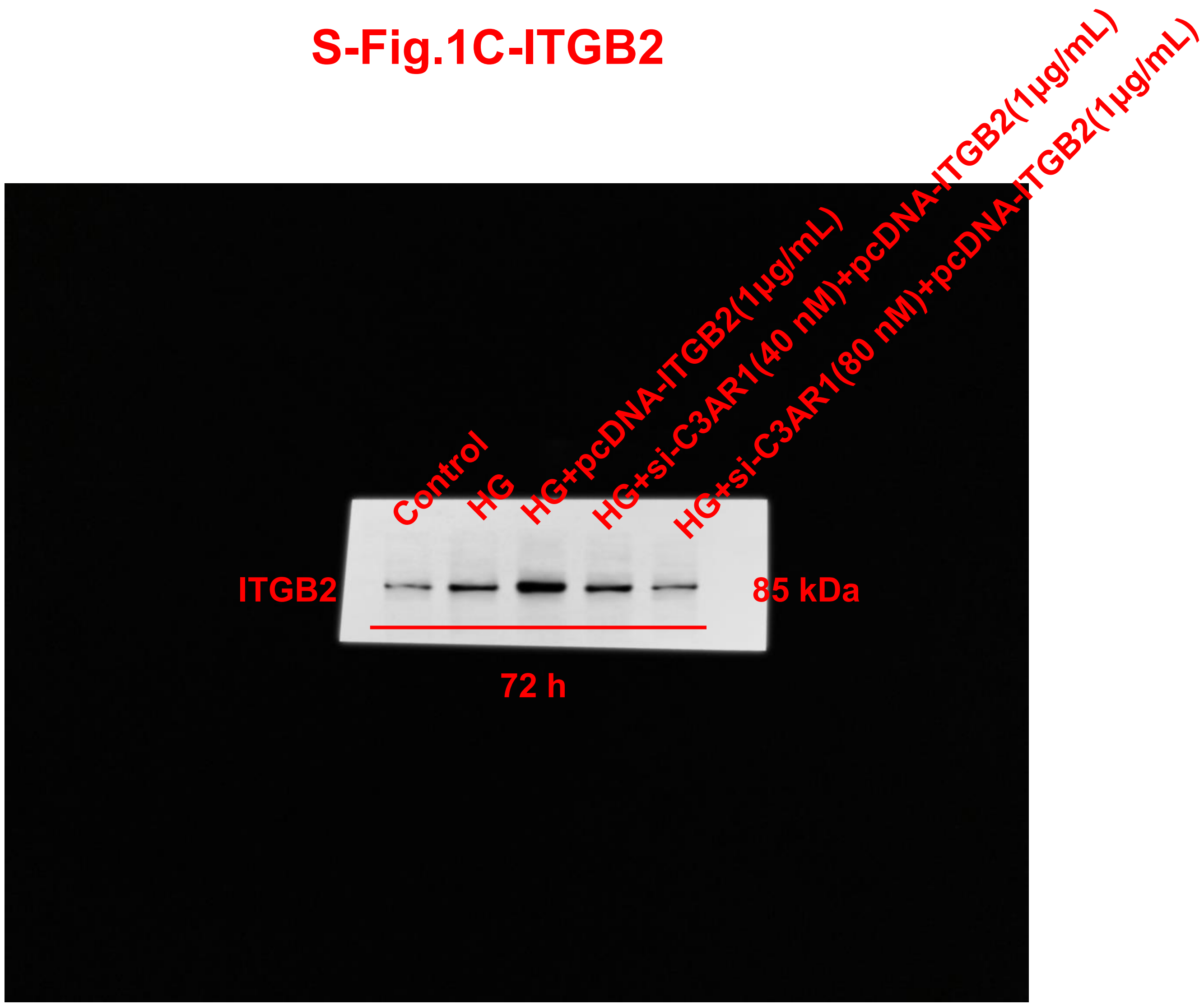

S-Fig.1C-β-actin

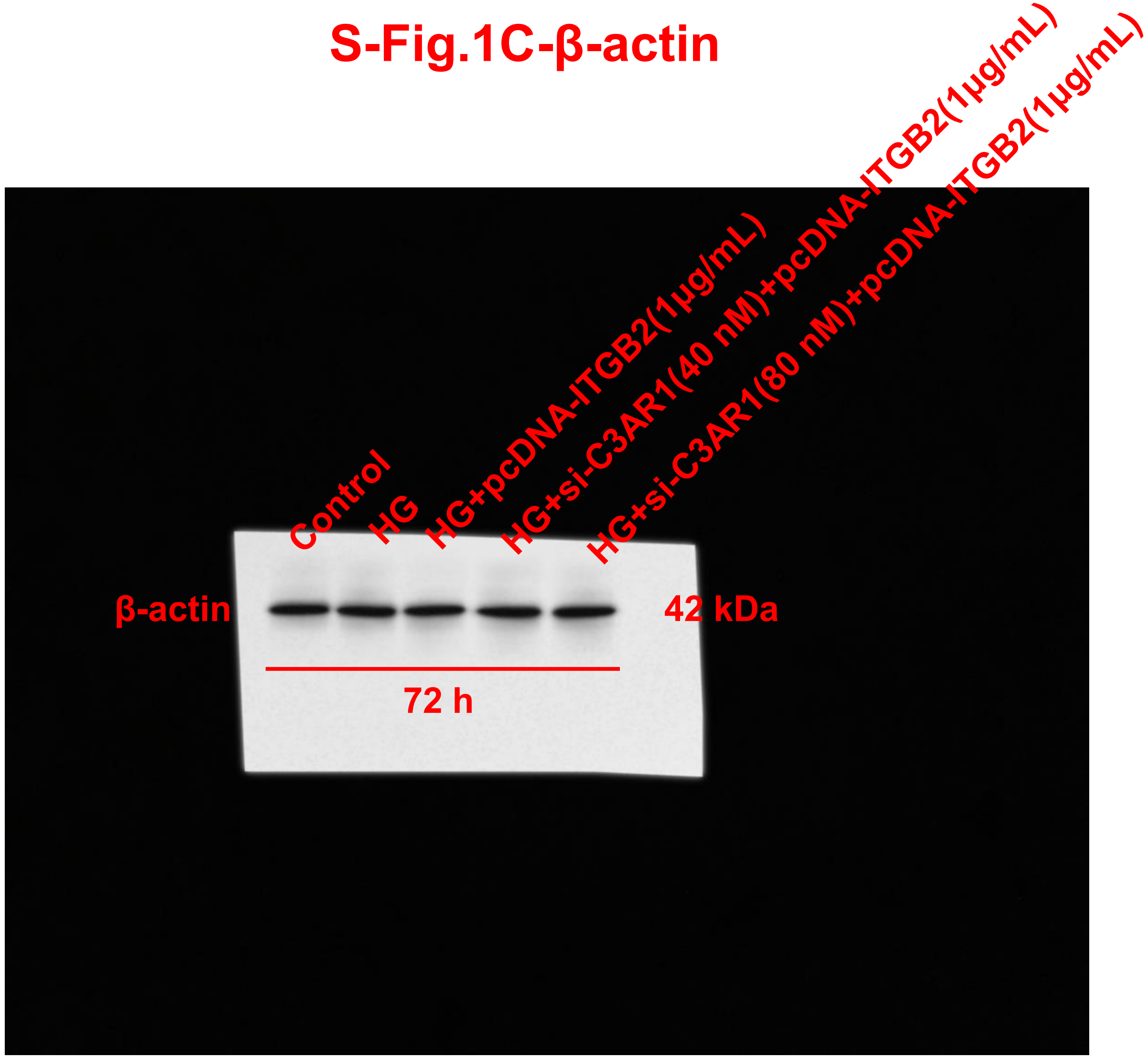

S-Fig.2A-C3AR1

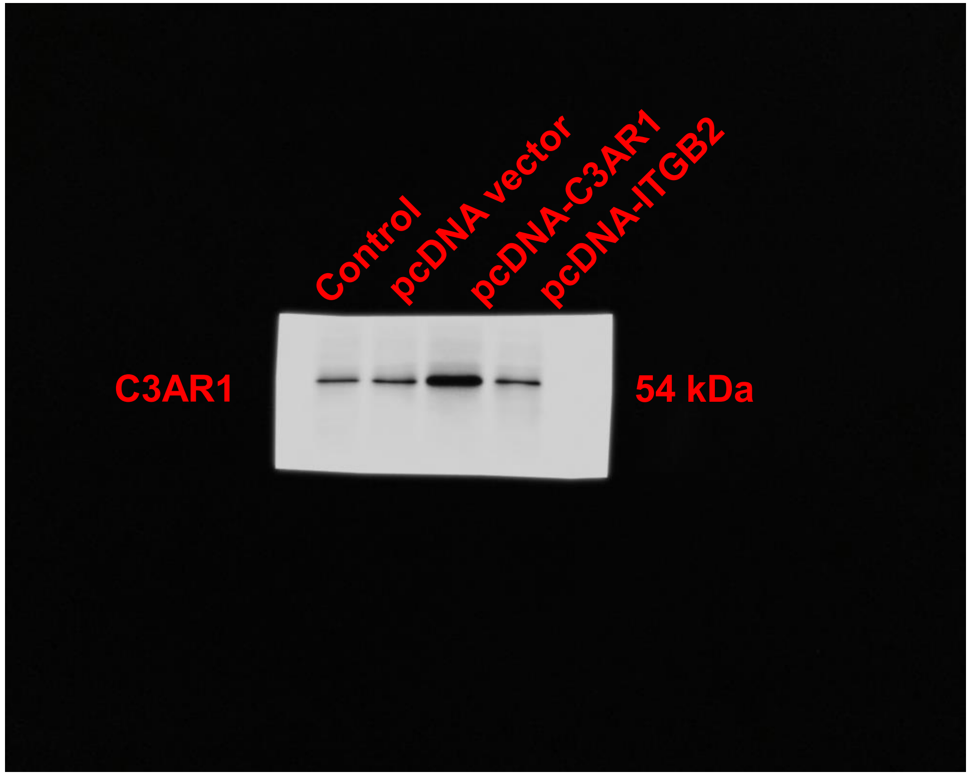

S-Fig.2A-ITGB2

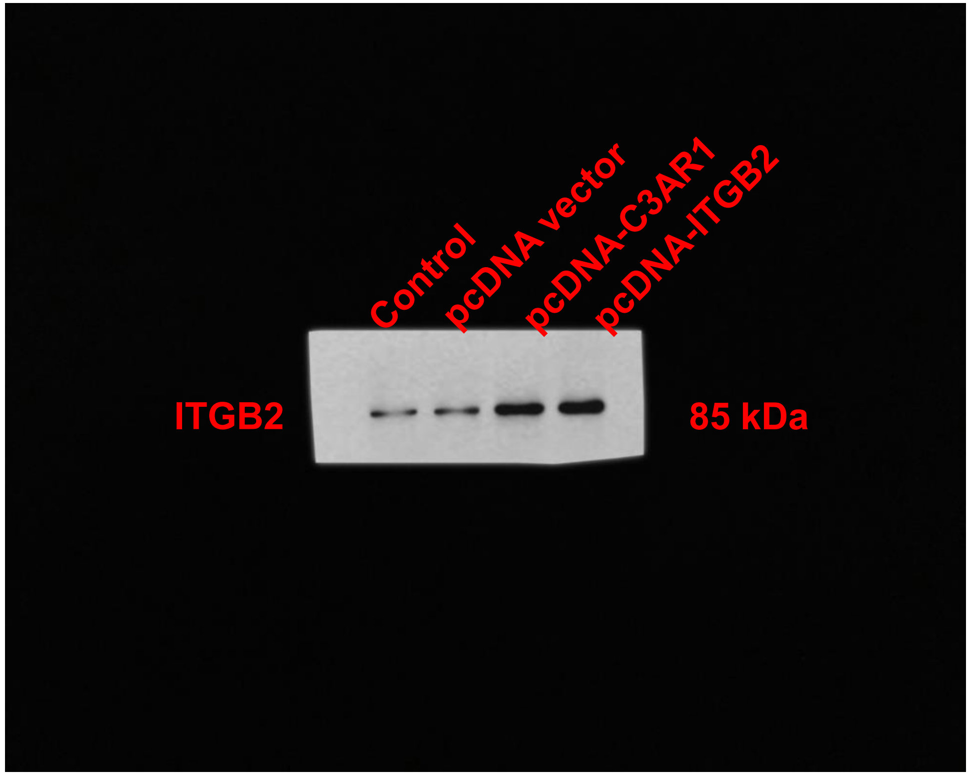

S-Fig.2A-β-actin

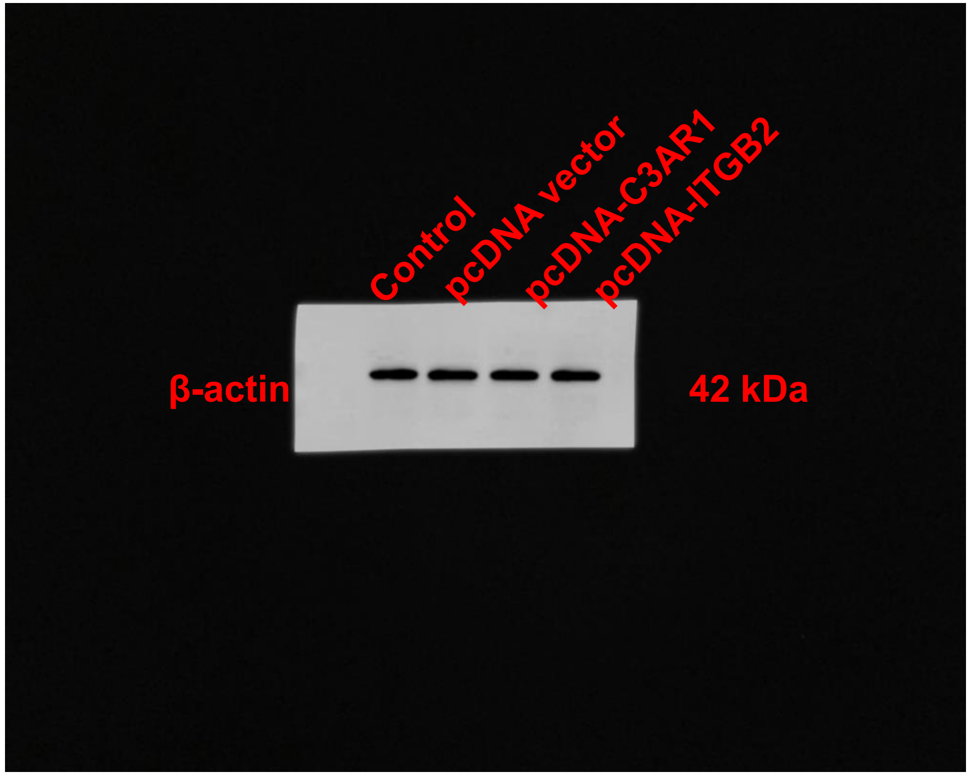

Supplement: S1 File — Original images for blot and gel. (PDF) [file pone.0331900.s004.pdf]
